# Genomics and metatranscriptomics of biogeochemical cycling and degradation of lignin-derived aromatic compounds in thermal swamp sediment

**DOI:** 10.1038/s41396-020-00820-x

**Published:** 2020-11-02

**Authors:** David J. Levy-Booth, Ameena Hashimi, Raphael Roccor, Li-Yang Liu, Scott Renneckar, Lindsay D. Eltis, William W. Mohn

**Affiliations:** 1 Department of Microbiology and Immunology, Life Sciences Institute, BioProducts Institute, The University of British Columbia, Vancouver, BC Canada; 2 Advanced Renewable Materials Lab, Department of Wood Science, BioProducts Institute, The University of British Columbia, Vancouver, BC Canada

**Keywords:** Biogeochemistry, Water microbiology, Freshwater ecology, Biogeochemistry

## Abstract

Thermal swamps are unique ecosystems where geothermally warmed waters mix with decomposing woody biomass, hosting novel biogeochemical-cycling and lignin-degrading microbial consortia. Assembly of shotgun metagenome libraries resolved 351 distinct genomes from hot-spring (30–45 °C) and mesophilic (17 °C) sediments. Annotation of 39 refined draft genomes revealed metabolism consistent with oligotrophy, including pathways for degradation of aromatic compounds, such as syringate, vanillate, *p*-hydroxybenzoate, and phenol. Thermotolerant *Burkholderiales*, including *Rubrivivax* ssp., were implicated in diverse biogeochemical and aromatic transformations, highlighting their broad metabolic capacity. Lignin catabolism was further investigated using metatranscriptomics of sediment incubated with milled or Kraft lignin at 45 °C. Aromatic compounds were depleted from lignin-amended sediment over 148 h. The metatranscriptomic data revealed upregulation of *des*/*lig* genes predicted to specify the catabolism of syringate, vanillate, and phenolic oligomers in the sphingomonads *Altererythrobacter* ssp. and *Novosphingobium* ssp., as well as in the *Burkholderiales* genus, *Rubrivivax*. This study demonstrates how temperature structures biogeochemical cycling populations in a unique ecosystem, and combines community-level metagenomics with targeted metatranscriptomics to identify pathways with potential for bio-refinement of lignin-derived aromatic compounds. In addition, the diverse aromatic catabolic pathways of *Altererythrobacter* ssp. may serve as a source of thermotolerant enzymes for lignin valorization.

## Introduction

Few geothermally influenced wetlands exist on Earth—geothermal hot springs that feed marshlands can be found in Yellowstone National Park (USA) [1–3], Great Rift Valley (Kenya), Iceland, and New Zealand [4]—fewer still are forested. The Liard River Hot Springs in northern British Columbia, Canada feature geothermally warmed springs (30–55 °C) with organic sediments and biomats that receive inputs from the surrounding mixed-forest vegetation. Based on these characteristics, we hypothesized that this environment harbors thermotolerant microbiota capable of degrading lignin-derived aromatic compounds.

Geothermally warmed systems are hot spots of chemoautotrophy and other oligotrophic adaptations [5, 6]. Dilute chemical resources may select for high-affinity and high-yielding chemoautotrophy. Specific examples include complete ammonia oxidation (Comammox) [7, 8], the conversion of cyanate and urea to ammonium [7, 9] and carboxydotrophy [10, 11]. Pathways for aromatic catabolism are also associated with oligotrophic bacteria, as with the mesophilic sphingomonad, *Sphingopyxis alaskensis* RB2256 [12]. Thermophilic mineralization of lignin-derived aromatic compounds is of both ecological and biotechnological interest [13–15]. Lignin is a recalcitrant plant heteropolymer synthesized from three phenylpropanoids: *p*-coumaryl (H), coniferyl (G), and sinapyl (S) alcohols. The degradation of lignin yields a variety of aromatic compounds. Due to the convergent organization of bacterial catabolic pathways, they are well suited to the biocatalytic conversion of these biomass-derived aromatics to fuels and chemicals [16–18]. The bacterial catabolism of S-lignin compounds, such as syringate, is primarily limited to aerobic sphingomonads [17, 19, 20] and anaerobic Firmicutes [21–24]. Few S-lignin-degrading thermophiles have been reported [24], and no aerobic, thermotolerant S-lignin degradation pathways have been elucidated. There are two main routes for degradation of syringate in bacteria [25, 26]: one via gallate using vanillate/3-*O*-methylgallate *O*-demethylase (LigM), and gallate dioxygenase (DesB), and one via 3-*O*-methylgallate carried out by protocatechuate 4,5-dioxygenase (LigAB) and 3-*O*-methylgallate 3,4-dioxygenase (DesZ). The later route can result in two different products with unique oxidation pathways [26]. Identifying enzymes capable of metabolizing lignin-derived aromatic compounds at elevated temperature would further biorefining and the move away from fossil feedstocks.

Herein, we used genome-resolved shotgun metagenomics and metatranscriptomics to characterize the phylogenetic and metabolic diversity of a thermal swamp. We exploited the negative, unimodal relationship between temperature and microbial diversity [27–29] to accomplish high-coverage genome reconstruction from the hot-spring microbiome. We then used metatranscriptomics to test the hypothesis that thermal swamp communities contained active microbial populations involved in aromatic catabolism. Genomic reconstruction revealed diverse chemoautotrophic metabolism in the thermal microbiome but only partially elucidated aromatic pathways for lignin-derived compounds, while metatranscriptomics and analytical chromatography provided evidence for thermotolerant catabolism of aromatic compounds. In particular, this study characterized transcriptomes from oligotrophic sphingomonads associated with degradation of S- and G-lignin at elevated temperature.

## Material and methods

### Field site and sampling

Liard River Hot Springs (59.431N, 126.1W) is a complex of carbonate-hosted springs that feed geothermally warmed meteoric waters to extensive swampland underlaid by sandstone and shale [30, 31] (Supplementary Fig. 1). The pools are surrounded by unique thermal meadow vegetation, and mixed forest containing *Populus tremuloides, Betula papyrifera*, and *Picea glauca*. Four hot springs were sampled on September 27, 2018: the Alpha (50–55 °C), Beta (30–35 °C), Delta (30–40 °C), and Epsilon (40–45 °C) pools. Alpha pool contains a recreational bathing area, and was previously measured to have a pH of 6.5–6.8 and concentrations of 0.9–1.0 mg l^−1^ O_2_, 180–187 mg l^−1^ HCO_3_, 554–592 mg l^−1^ SO_4_, 0.4–33.8 mg l^−1^ H_2_S, < 1 mg l^−1^ NO_3_, and <0.005 mg l^−1^ Fe [31, 32]. Gas in the water column was primarily N_2_ (93.9%) with trace CO_2_ (4.2%) and methane (0.3%) [32]. Water cools as it traverses the marshland via the Epsilon and Alpha riverine outflows to about 17 °C at our final sampling location (Cool). In each pool, three replicate ~500 ml bulk samples of sediment were removed with a syringe pump into autoclaved 1 l Nalgene^®^ bottles. Bottles were filled to the brim with spring water, and the pump was rinsed once with 70% ethanol and thrice with autoclaved distilled water between each sample. In addition, ~5 ml from each sampling location was placed in a Falcon^®^ tube and immediately frozen on dry ice for DNA extraction.

### DNA extraction, library preparation, and sequencing

DNA was extracted twice per sample, using 0.25 g sediment, soil, or biofilm, with NucleoSpin Soil kits (Machery-Nagel, Düren, Germany) then pooled. DNA quality was assessed with a 1% agarose gel and concentration was measured with Qubit dsDNA high-sensitivity assays (ThermoFisher, Waltham, USA). 1 ng of DNA from three replicates of selected sediment samples (Cool, Beta, Epsilon) was prepared for shotgun sequencing on one NextSeq550 (Illumina) run in High Output mode using NexteraXT library preparation (Illumina, San Diego, USA). Additional sampling and 16S rRNA gene sequencing are described in the Supplementary Methods.

### Lignin incubations

2 g of sediment from Epsilon, the warmest undisturbed pool, was incubated in 5 ml of M9-Goodies minimal medium [33, 34] with 2 mg of vanillin (VAN), Eucalyptus milled-wood lignin (EMWL) (see Supplementary Methods), eucalyptus kraft lignin (EKL), or coniferyl alcohol dehydrogenase-polymer model lignin (DHP). Eucalyptus wood chips and EKL were provided by Suzano Canada. DHP was synthesized as in [35]. Control incubations were conducted with no exogenous carbon. All incubations took place in 50-ml sealed serum bottles at 45 °C and 150 RPM mixing in the dark. During incubations, substrate-induced respiration (SIR) was measured by adding 1 ml air to the vial headspace with a 1-ml glass syringe, mixing, and removing 1 ml headspace, which was manually injected into a 5890 Series II gas chromatograph (Agilent Technologies, Santa Clara, USA) equipped with a flame ionization detector and methanizer, and quantified against CO_2_ standards (Praxair, Danbury, USA). Three separate incubations for each substrate were destructively sampled at 0, 48, 96, and 148 h, except for VAN, which was sampled only at 24 h. At each time point, samples were centrifuged at 4 °C for 5 min at 18,000 × *g*, and the supernatant was stored at −80 °C prior to high-pressure liquid chromatography (HPLC). RNA was immediately extracted from the remaining sediment.

### RNA extraction, library preparation, and sequencing

Approximately 0.5 g of sediment was separated into 2-ml screw-top microtubes containing 740 µl 0.1 M Na_2_PO_4_-NaH_2_PO_4_ pH 7.3, 60 µl 10% SDS and 800 µl 25:24:1 phenol:chloroform:isoamyl alcohol (Sigma Aldrich, St. Louis, USA), modified from [36]. RNases from thermophilic organisms are highly resistant to inhibition or denaturation. To recover high-quality RNA from thermophilic communities, 100 µl of 200 mM ribonucleoside vanadyl complex (RVC) (Sigma Aldrich) was added to the extraction buffer [37, 38]. RVC was essential for recovery of RNA from these samples (Supplementary Fig. 2A). Bead beating at 5.5 m/s for 30 s (twice) was followed by centrifugation for 5 min at 4 °C and 18,000 × *g*. The supernatant was added to 800 µl of chloroform:isoamyl alcohol (Sigma Aldrich), mixed by hand, and centrifuged again. The supernatant was purified using the RNeasy^®^ Mini Kit (Qiagen), and DNA was removed using Turbo DNase (ThermoFisher). RNA concentration was measured using the Qubit™ RNA HS assay (ThermoFisher), and purified RNA stored at −80 °C for <1 week. RNA integrity was measured using an Agilent 2100 Bioanalyzer (Agilent Technologies) before and after rRNA removal with RiboMinus™ Transcriptome Isolation Kit, bacteria (ThermoFisher). Sequencing libraries were prepared using SuperScript™ Double-Stranded cDNA synthesis (ThermoFisher) and NexteraXT (Illumina). Twelve 100-bp paired-end libraries were sequenced per NextSeq550 (Illumina) High Output run.

### Lignin analysis

Lignin substrates were analyzed using ^13^C nuclear magnetic resonance (NMR) and 2D heteronuclear single quantum coherence (HSQC) NMR analysis [39–41]. Full details are provided in Supplementary Methods.

### Liquid chromatography

Monoaromatic compounds in incubation supernatants were analyzed by HPLC against 2,6-dimethoxy-1,4-benzoquinone, vanillic acid, syringic acid, VAN, and syringaldehyde standard curves obtained by injecting between 1 and 20 μM of authentic standards in 50 mM sodium phosphate. Full details are provided in Supplementary Methods.

### Assembly of genomes from metagenomes

Shotgun metagenome libraries were quality filtered and adapter trimmed using Trimmomatic 0.38 with default settings [42] prior to MegaHit 1.1.3 co-assembly with “meta-large” presets [43]. Contig open reading frames (ORFs) were predicted using Prodigal 2.6.3 [44]. Taxonomy of each ORF was annotated using Kaiju 1.7.2 [45], which, in turn, was used to calculate contig consensus taxonomy if 50%+1 of contig ORFs shared taxonomic identity. Contig coverage was calculated with BBMap 38.22 (https://sourceforge.net/projects/bbmap/) prior to binning with Metabat2 [46]. CheckM 1.0.13 [47] was used to identify phylogenetic markers and calculate bin completeness, contamination, and heterogeneity. High-quality MAGs were manually refined (Supplementary Methods) and CheckM was re-run on the resulting refined genomes, followed by taxonomic classification and placement in whole-genome phylogenetic trees using GTDB-Tk [48] (github.com/Ecogenomics/GTDBTk). A metatranscriptome co-assembly was performed as above but using only 25-mers and binned without the support of coverage calculations.

### Metabolic pathway annotation

Genes were annotated using a set of previously compiled Pfam and TIGRFAMs profile HMMs representing enzymes involved in biogeochemical cycling and energy metabolism [49–51], as well as Kofamscan profile HMMs [52]. Hits for ammonia oxidation (*amoA*) and nitrite oxidoreductase genes (*nxrA*) were validated by phylogenetic placement. Additional archaeal ammonia oxidation genes were identified using local alignment against *Nitrososphaera viennensis* EN76 *amoABC* [53]. Carbohydrate-active enzyme (CAZy) gene annotations were defined using dbCAN2 [54] with an *E* value of 1e−60. To facilitate the identification aromatic compound degradation and anoxygenic photosystem genes, custom thresholds were set for 25 KEGG HMMs (Supplementary Data 1, Supplementary Methods).

### Calculating genomic abundance

Quality-filtered read files from metagenome and transcriptome libraries were aligned to the 39 refined genomes with BBMap. To capture potential biodiversity missed by the assembly approach, unmapped reads were subsequently aligned with 351 remaining contig bins and 24,668 reference genomes [48]. Genomes with >1% coverage were included in subsequent genomic analysis. To visualize the distribution of each genome across temperatures, feature scaling was used to normalize counts in each metagenome library between 0 and 1 for all genomes independently.

### Statistical analysis

Comparison of OTU richness, assembly coverage, and genome richness across sequencing libraries was made using unpaired *t*-tests in R v3.6.2 [55] with a significance cutoff of *p* < 0.05. Headspace CO_2_ was also compared with unpaired *t*-tests. Genomic and transcriptomic bins were analyzed for transcription levels in incubations with DHP (48 h), EKL (96 h), EMWL (168 h), VAN (24 h), and NC (96 h), coinciding with sampling times when maximum RNA was recovered (Supplementary Fig. 2B). Transcript counts from metatranscriptome libraries were compared as transcripts per million with generalized linear models (GLM) and the *multcomp* Tukey-HSD implementation in R against NC controls. Differential gene expression was analyzed across metatranscriptomes using DESeq2 [56], using log_2_ fold change (L_2_FC) values, and a significance cutoff of *p*_adj_ < 0.05 following false-discovery rate correction.

## Results

### Influence of temperature on hot-spring microbial diversity

**Fig. 1:**
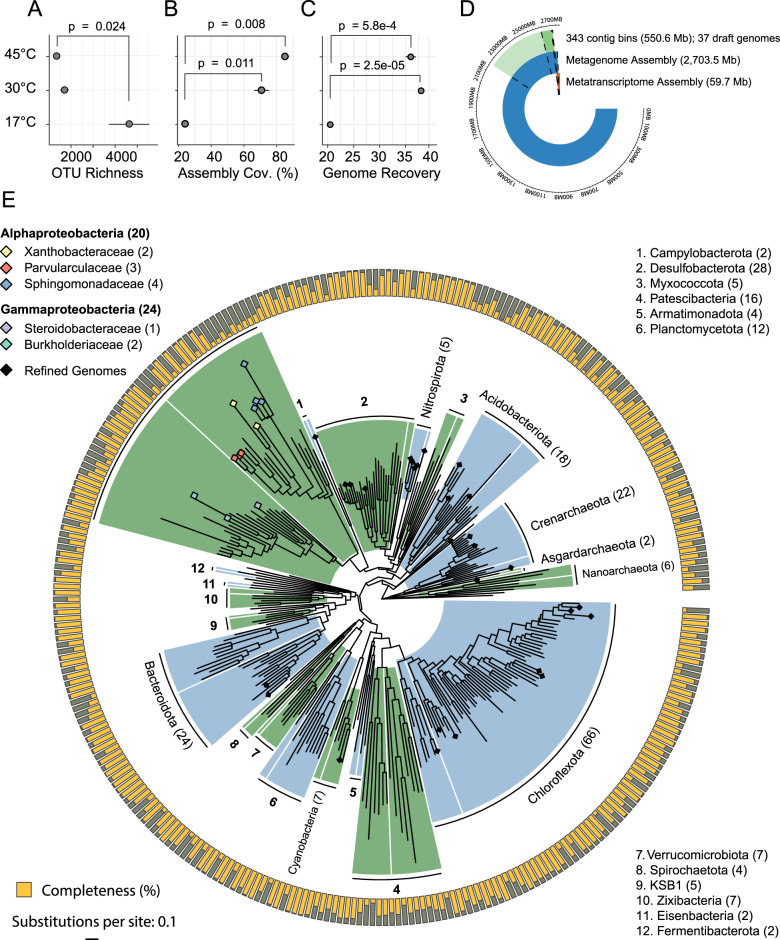
Diversity, assembly, and phylogeny of a thermal swamp community. **A** Richness of 16S rRNA gene OTUs in samples from Epsilon (EPS, 45 °C) and Beta (BETA, 30 °C) hot springs and from mesophilic sediment (COOL, 17 °C) (*n* = 3). **B** Assembly coverage across samples. **C** Number of recovered refined genomes across samples. **D** Length of the full metagenome assembly in Mb compared with metagenome-assembled genome (MAG) bins, refined (draft) genomes, and the metatranscriptome assembly. **E** Whole-genome phylogeny of the MAGs shaded by phylum. Number of bins in each phylum indicated in parentheses. The outer ring shows bin completeness assessed with CheckM 1.0.13. Tree scale represents substitutions per site. Bins belonging to proteobacterial taxa implicated in degradation of lignin-derived aromatic compounds are highlighted with shaded diamonds.

We first characterized community-level patterns across hot-spring samples using 16S rRNA amplicon sequences. Prokaryote community structure differed by sample type (sediment, biomat, soil) (*p* = 0.001) and temperature (*p* = 0.001) following PERMANOVA (Supplementary Fig. 3A). Shannon–Weiner diversity was negatively correlated with temperature (*R*^2^ = 0.73, *p* < 0.001) (Supplementary Fig. 3B). Generally, we saw lower 16S rRNA OTU richness (Fig. 1A) in warmer samples.

### Assembly of genomes from thermal sediment metagenomes

We then sequenced nine metagenomic libraries, three each from two hot springs receiving woody carbon inputs (Epsilon (45 °C), Beta (30 °C)) and three from mesophilic riverine samples (Cool (17 °C)). In total, 168 million paired-end reads (41.65 gigSuzano abases (Gb)) passing quality filtering were assembled into 2.7 Gb of contigs (Supplementary Data 2). About 20% of the combined assembly was binned into 343 MAGs based on GC content, tetranucleotide frequency, sequencing coverage across samples, and presence of SCGs [50–52] (Fig. 1D). Metagenome assembly coverage was significantly higher for 30 °C (*p* = 0.011) and 45 °C samples (*p* = 0.008) than for the 17 °C samples following unpaired *t*-tests (Fig. 1B). All pools clustered distinctly following PCoA ordination (Supplementary Fig. 3C). Likewise, refined genome recovery was significantly greater for the 30 and 45 °C samples (*p* < 0.001) than for the 17 °C samples (Fig. 1C).

### Phylogenetic distribution of assembled genomes

**Fig. 2:**
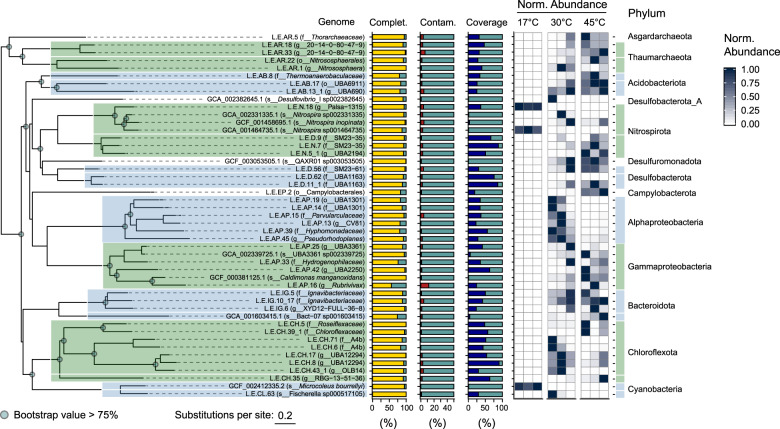
Refined genome phylogeny, assembly statistics, and abundance in Epsilon (45 °C) and Beta (30 °C) hot springs and mesophilic sediment (17 °C) metagenomic libraries. Whole-genome phylogeny assessed as in Fig. 1. Tree scale represents estimated site divergence and green dots indicate branching calculated with bootstrapped confidence > 75%. Completeness (yellow) and contamination (red) were estimated with CheckM 1.0.13. Genome abundance calculated by alignment of quality-filtered reads against refined genomes and GTDB reference genomes using BBMap 38.22 and retaining targets > 0.5% coverage (blue). Abundance counts normalized using feature scaling between 0 and 1 for each genome.

Sixty-six (19%) of MAGs were placed into phylum *Chloroflexota* (Fig. 1E and Supplementary Data 3), including families *Roseiflexaceae* (L.E.CH.5) and *Chloroflexaceae* (L.E.CH.39) (Fig. 2). Forty-four MAGs (13%) were placed into the *Proteobacteria*. In particular, two *Burkholderiaceae* and four *Sphingomonadaceae* MAGs were identified. Notably, two *Asgardarchaeota* MAGs were recovered, representing potentially novel genera in the class, *Lokiarchaeia* (Supplementary Data 3).

MAGs can contain erroneously binned sequences that can lead to faulty ecological interpretation. While MetaBat2 performs favorably compared to other binning algorithms [46], we nonetheless manually refined 39 MAGs with contamination < 5% and completeness > 80% (unless otherwise noted) into our final “refined genome” data set (Supplementary Fig. 4). Subsequent analyses focused on the refined genomes because: (1) they are taxonomically representative of the larger MAG data set; (2) many MAGs have incomplete or erroneous assembly; and (3) the refined genomes are the most abundant in the metagenome libraries based on read coverage. Only one *Burkholderiaceae* genome (*Rubrivivax* sp. L.E.AP.16) was included in the refined genome pool, despite a completion estimate of 56%, due to its unique metabolic capacity and possible thermotolerance. *Sphingomonadaceae* MAGs had a mean completeness of only 28%, and none were included in the analysis of refined genome.

### Environmental distribution of refined genomes

The temperature at which each refined genome was found to have the highest relative abundance was used to infer their potential thermotolerance. We quantified the relative abundance of the refined genomes across a thermal gradient by aligning quality-filtered reads from shotgun metagenome libraries to refined genomes. We additionally assessed genomes potentially missed by our approach by aligning remaining reads against a collection of species-level reference genomes. Both refined and reference genomes were used to evaluate relative abundance and diversity across thermal samples. Comammox *Nitrospira* genomes were abundant at 17 °C (see Supplementary Figs. 5 and 6 for Amo and Nxr phylogenies), highlighting the oligotrophy of this environment. Twelve of 41 genomes used in this analysis were most abundant at 30 °C (Fig. 2). These included many of the *Proteobacteria*, excluding L.E.AP.16, and the manganese-oxidizing thermophile, *Caldimonas manganoxidans* [57]. Two of the most abundant genomes at 45 °C were the Chloroflexota L.E.CH.5 and L.E.CH.39, at 9.8% ± 10.9% and 7.1% ± 7.6%, respectively.

### Inferring genome metabolic potential across temperature ranges

**Fig. 3:**
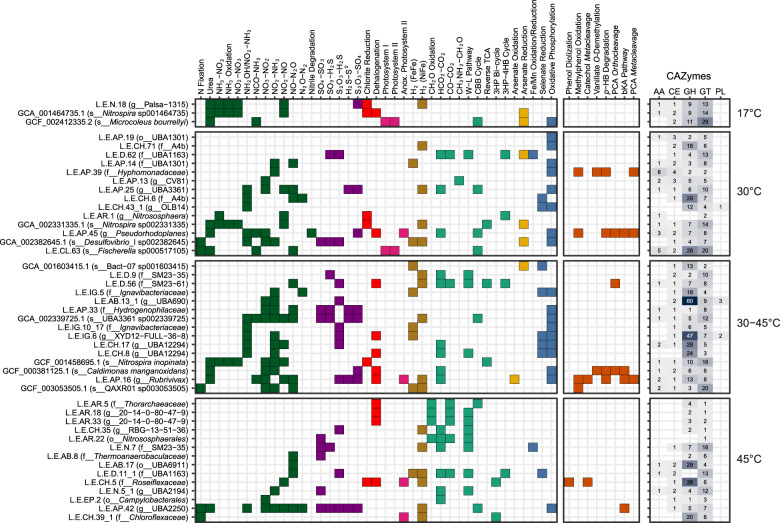
Genome functional annotation. Annotation of key genes and pathways involved in energy and carbon metabolism used kofamscan 1.1.0 profile hidden Markov models (HMMs) and select PFAM and TIGRFAM HMMs. Nitrogen cycling genes are shown in green, sulfur cycling in purple, dehalogenation in red, photosynthesis in pink, H_2_-oxidation in gold, carbon fixation in teal, arsenate metabolism in yellow, oxidative phosphorylation in blue, and aromatic degradation in orange. Number of CAZymes in each class were determined using dbCAN2. W–L Wood–Ljungdahl, 3HP 3-hydroxypropionate, 4-HB 4-hydroxybutyrate, *p*-HB *p*-hydroxybenzoate, PCA protocatechuate, bKa beta-ketoadipate, GH glycoside hydrolases, GT glycosyltransferases, PL polysaccharide lyases, CE carbohydrate esterases, AA auxiliary activities.

The metabolic capacity of each genome was inferred using profile HMMs from select Pfam [50], TIGRFAMs [49, 51], CAZy [58], and KEGG [52] protein families (Supplementary Data 1), as well as local alignment against the UniRef90 database [59]. Metabolic pathways were positively annotated when they contained essential enzymes such as monooxygenases or dioxygenases, and also contained at least 50%+1 of all genes identified using the above pHMMs (Fig. 3). Gene-level annotation is provided in Supplementary Fig. 7 and Supplementary Data 4.

**Fig. 4:**
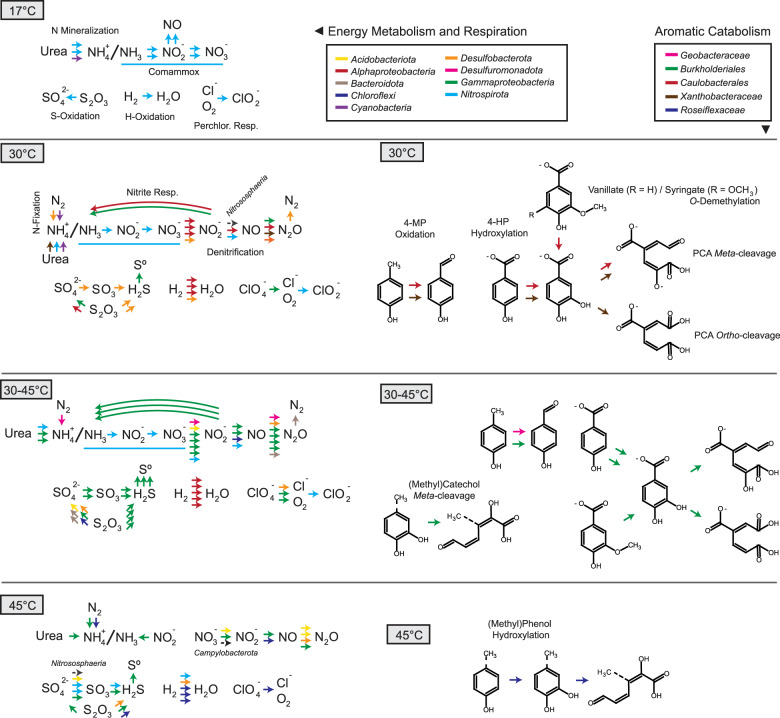
Predicted energy metabolism, respiration, and aromatic degradation pathways encoded in thermal swamp genomes shown in Fig. 3.

Multiple refined genomes were found only at 30 or 45 °C, while 14 were similarly abundant in both pools (Fig. 2). The genomes from three temperature categories (30, 45, and 30–45 °C) encoded similar biogeochemical cycling capacity, with differences in the taxonomy of microorganisms involved in nitrogen, sulfur, hydrogen, and halogen metabolism (Figs. 3 and 4). At 30 °C, a phylogenetically diverse group of genomes encoded transformation of nitrogen, including a cyanobacterium, *Fischerella* sp. (L.E.CL.63), which encoded capacity for both N-fixation and urea mineralization. A *Nitrososphaera* genome (L.E.AR.1), containing a full complement of archaeal ammonia-monooxygenase genes (Fig. 3 and Supplementary Figs. 5 and 6), was maximally abundant at 30 °C. The abundance of Comammox *Nitrospira* sp. and group I.1b Thaumarchaeota ammonia-oxidizing archaea indicates highly oligotrophic conditions, i.e., [NH_3_ + NH_4_^+^] < 5 µM [7]. The capacity for dissimilatory nitrogen respiration was encoded by two alphaproteobacterial genomes, L.E.AP.45 and L.E.AP.39, which also contained pathways for aerobic aromatic catabolism via *meta-* and *ortho-*cleavage of protocatechuate (Fig. 4), indicating the potential for facultative anaerobic respiration and aerobic biomass decomposition.

The genomes that showed equivalent abundance at 30 and 45 °C (30–45 °C) may represent thermotolerant organisms. *Burkholderiales* genomes of L.E.AP.16 (*Rubrivivax* sp.) and *C. manganoxidans* encoded aromatic degradation capacity though the catechol *meta*-cleavage and protocatechuate *meta-* and *ortho-*cleavage pathways (Figs. 3 and 4). The capacity for vanillate degradation was encoded in two-component vanillate *O*-demethylase (*vanAB*) in *C. manganoxidans*, rather than in the tetrahydrofolate-dependent vanillate/syringate *O*-demethylase (*ligM*/*desA*) found in a *Caulobacterium* genome from 30 °C. The *vanAB* genes were not detected in the L.E.AP.16 genome, despite expression of their homologs in *Rubrivivax* transcriptomes (see below). The *Burkholderiales* genomes abundant in the 30–45 °C range also showed substantial carbohydrate degradation capacity based on the presence of high numbers of glycoside hydrolases (GHs) (Fig. 4 and Supplementary Fig. 8).

Genomes found only 45 °C may represent moderate thermophiles. The *Chloroflexota* genome L.E.CH.5 encoded the ability to hydroxylate alkylated phenols with an actinobacterial-like two-component monooxygenase [16], in addition to catechol *meta*-cleavage. In total, thermotolerant and thermophilic *Burkholderiales* genomes encoded a large portion of the biogeochemical cycling and aromatic degradation at elevated temperatures, with a range of possible electron donors and acceptors allowing substantial metabolic flexibility.

### Lignin incubations

**Fig. 5:**
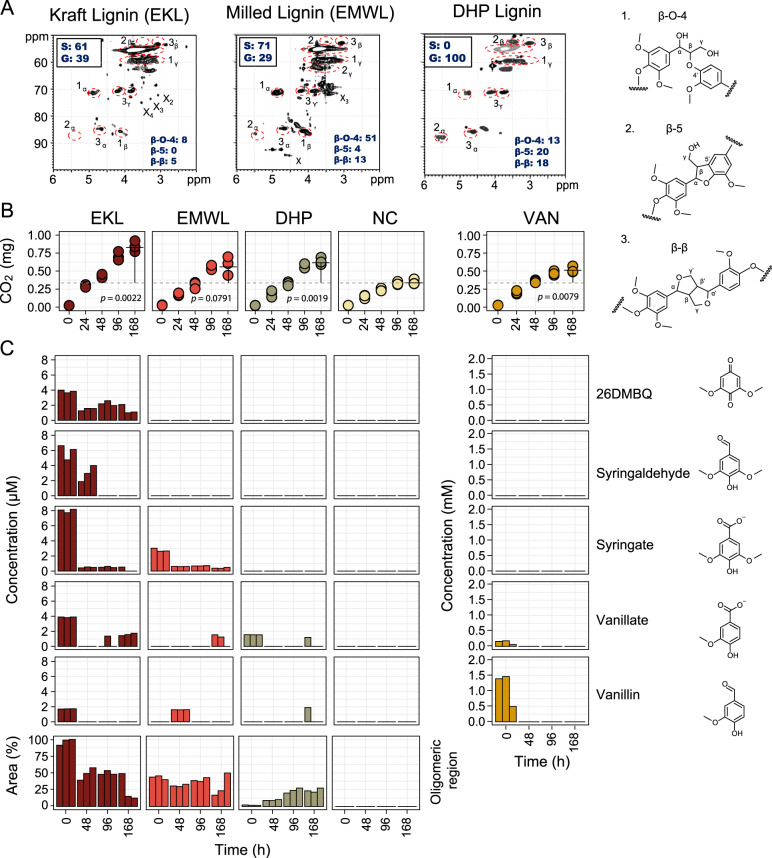
Lignin structure and aromatic compound concentrations in Epsilon pool sediment incubations. **A** 2D HSQC NMR spectra of initial lignin substrates showing bond types and proportions. Values for bond types and syringyl (S) and guaiacyl (G) subunits (^13^C NMR) are normalized per 100 aromatic units. Examples of each bond type are provided (1–3). **B** CO_2_ evolution in incubation vial headspace. Total CO_2_ compared with no exogenous carbon (NC) controls at 168 h using Student’s *t* test. **C** Concentrations of five monoaromatic compounds in triplicate incubations over 168 h during incubation experiments. Bars representing lignin-derived aromatic oligomers scaled to percent of max peak area. 26DMBQ 2,6-dimethoxy-1,4-benzoquinone, VAN vanillin.

To characterize thermotolerant modification of lignin and the catabolism of lignin-derived aromatic compounds, we incubated Epsilon pool (45 °C) sediment with one of three preparations of lignin (EMWL, EKL, and DHP lignin), VAN, or a no-carbon control. Epsilon sediment was chosen as it was the warmest undisturbed sampling location. 2D HSQC NMR analysis revealed that EMWL, generated by extracting lignin from enzymatically treated, milled Eucalyptus wood chips, was much less modified than EKL, generated by the Kraft process (Fig. 5A). Specifically, EMWL contained 51 β-aryl ether bonds per 100 aromatic subunits, while EKL contained only eight. Further, β-5 bonds were detected in EMWL but not EKL, and EMWL contained a higher S:G ratio (7:3) than EKL (6:4). EMWL, and to a lesser extent EKL, contained residual carbohydrates (annotated with an “X” in Fig. 5A). The synthetic DHP lignin contained a higher β-5 ratio than EMWL and, consistent with its synthesis, no carbohydrate and only G-lignin units. Finally, EMWL and EKL contained small amounts of monoaromatic compounds, such as vanillate and syringate, as well as aromatic oligomers, while DHP lignin was relatively free of these compounds. EKL specifically contained about three times as much S-lignin-derived monoaromatics than G-lignin-derived compounds.

SIR during lignin incubations was monitored by quantifying CO_2_ in the headspace. CO_2_ production significantly increased by 168 h in the presence of EKL (240%, *p* = 0.0022), EMWL (170%, *p* = 0.079), DHP (180%, *p* = 0.0019), and VAN (150%, *p* = 0.0079) relative to NC control incubations (Fig. 5B). The aromatic constituents of the incubation supernatant were analyzed with HPLC. At time zero, we detected monoaromatic compounds in the lignin-amended samples, and a large peak provisionally assigned to aromatic oligomers based on compound size. The area of this large peak decreased ~50% by 48 h. In EKL, syringate concentrations were reduced by 94% after 48 h, syringaldehyde disappeared from the supernatant after 96 h, and VAN was completely removed after 48 h.

The initial concentrations of monoaromatic compounds in lignin-amended samples (*t* = 0) were in the low µM range. As this is much lower than the concentration of VAN (~1.4 mM), their presence alone does not explain the measured respiration, as the lignins induced more CO_2_ production than VAN. As SIR is a very broad measure of metabolic activity, we also cannot rule out the impact of the potential toxicity of VAN [60] on the CO_2_ production, or the possible effect of added lignins on the degradation of endogenous carbon. Vanillate and VAN were only detected after several days of incubation with EMWL and DHP, consistent with depolymerization. However, further analysis of lignin substrates would be required to confirm the possibility of depolymerization. Such analysis was not possible in the current study as the samples were sacrificed to obtain RNA yields suitable for metatranscriptomics analysis described in the next section.

### Metatranscriptomics of lignin-incubated thermal sediment

**Fig. 6:**
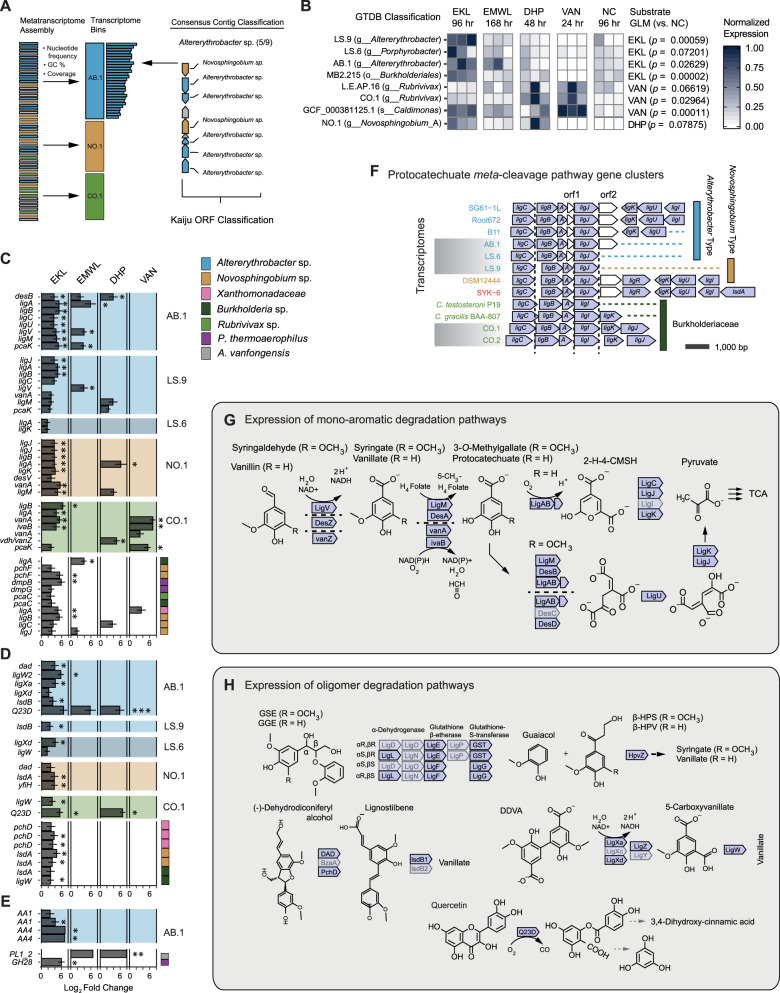
Metatranscriptomics of hot-spring sediment incubated with 0.02% (m/v) DHP, eucalyptus kraft lignin (EKL), Eucalyptus milled-wood lignin (EMWL), vanillin (VAN), and control incubations with no exogenous carbon (NC). **A** Strategies for taxonomic identification and binning of metatranscriptome contigs. **B** BBMap read mapping to MAGS (MB2), refined genomes (L.E.), GTDB reference genomes (GC), and binned transcriptome assemblies (AB, CO, LS). Mapped read counts per bin were normalized using length of the contig assembly and depth of sampling. *p* values generated from the *multcomp* implementation of Tukey-HSD pairwise-GLM analysis of normalized counts for lignin substrate shotgun metatranscriptome libraries against NC libraries (*p* < 0.05 shown). **C** Transcripts putatively involved in catabolic pathways for methoxylated monoaromatics (vanillate/syringate). Taxonomy determined using Kaiju and GTDB-Tk classification of assembled transcriptomes as shown in (**A**). Results of DESeq2 differential expression (DE) analysis against NC libraries provided as log2 fold change values if (*p*_adj_ < 0.1). Transcripts that are differentially expressed with *p*_adj_ < 0.05 are denoted with an asterisk (*). **D** Transcripts putatively involved in aromatic oligomer degradation. **E** CAZyme transcripts. **F** Proteobacterial protocatechuate *meta*-cleavage (*lig*) gene clusters identified in genome and transcriptome bins. Clusters are aligned to the start position of the *ligJ*/*I* reading frame. **G** Vanillate and syringate *meta*-cleavage by *des*, *van*, and *lig* gene products in the eucalyptus kraft lignin (EKL) transcriptomes. **H** Degradation of guaiacylglycerol-β-guaiacyl ether (GGE), 5,5′-dehydrodivanillate (DDVA), and other oligomeric compounds. Lightened genes were not detected in transcriptome.

To understand how thermotolerant communities can modify lignin, and to identify mineralization pathways for LMW aromatic compounds, we sequenced samples from all incubation time points with >100 ng total RNA. Transcriptome contig taxonomy was assessed with Kaiju (“Material and methods”), as well as analysis of SGCs in MetaBat2 “transcriptomes” (Fig. 6A). Taxonomically classified transcriptomes were added to the refined and reference genomes for read alignment and quantification as above. Transcriptomic reads mapped to 37 genomes and 6 transcriptomes. GLM analysis with Tukey post hoc testing indicated that four bins had higher normalized read-mapping rates in EKL relative to controls (Fig. 6B). These included two *Altererythrobacter* transcriptomes, LS.9 and AB.1, and one *Burkholderiales* genome, MB2.215. Two *Burkholderiales* showed higher overall gene expression in VAN incubations: the *Rubrivivax* transcriptome CO.1, and the *C. manganoxidans* genome.

Differential expression analysis was performed with DESeq2 to determine transcript-level expression patterns during lignin incubations (Fig. 6C–E). Twenty-six transcripts in aromatic degradation pathways were found to be significantly upregulated on EKL using a cutoff of *p*_adj_ < 0.05, compared to only nine in EMWL and three in VAN incubations (Fig. 6C). Several upregulated transcripts were identified as homologs of syringate or vanillate degradation pathway genes (Fig. 6G). Phylogenetic analysis (Supplementary Figs. 9–14) and syntenic conservation (Fig. 6F) of the protocatechuate *meta*-cleavage pathway genes were assessed to validate these assignments. Transcripts assigned to this pathway were found in AB.1, LS.6, LS.9, NO.1, CO.1, and CO.2 transcriptomes (Supplementary Data 5).

The protein phylogenies derived from metagenomic and metatranscriptomic sequences for vanillate *O*-demethylase (LigM), syringate *O*-demethylase (DesA), protocatechuate 4,5-dioxygenase α (LigA) and β (LigB) subunits were assessed by sequence alignment and phylogenetic tree estimation (Supplementary Figs. 9–14). The AB.1 and LS.9 bins encoded all of these enzymes, except LigM, which was only encoded in AB.1. Differences existed between these two bins, despite both being classified as *Altererythrobacter* on the basis of SCG phylogeny. The two bins contained identical *desA* genes; however, LS.9 encoded protocatechuate 4,5-dioxygenase subunits that clustered with those of SYK-6, while AB.1 encoded homologs that clustered with those of *Altererythrobacter*. Furthermore, the presence of an uncharacterized ORF directly upstream of the *ligA* was detected in only *Altererythrobacter* reference genomes and AB.1, while the corresponding genes in LS.9 were syntenous to those of SYK-6 and *N. aromaticivorans* (Fig. 6F).

The sphingomonads SYK-6 and *N*. *aromaticivorans* have been widely investigated for their ability to depolymerize model lignin compounds [61, 62]. We identified potential homologous depolymerization systems in moderately thermophilic *Altererythrobacter* and *Novosphingobium* transcriptomes (Fig. 6D). Two putative *Altererythrobacter ligX* homologs (L_2_FC = 4.1, *p*_adj_ = 0.03; L_2_FC = 2.2, *p*_adj_ = 0.06) and one putative *ligW* (L_2_FC = 5.8, *p*_adj_ = 0.02) implicated in the catabolism of the methoxylated biphenyl compound 5,5′-dehydrodivanillate (DDVA) were upregulated on EKL after 96 h. The NO.1 and LS.9 transcripts encoding *ligW* sequences clustered with SYK-6, *Novosphingobium*, and *Altererythrobacter* homologs following phylogenetic placement (Supplementary Fig. 13). However, *ligXc* and *ligY* homologs were not identified (Fig. 6H). Several glutathione *S*-transferases (GSTs) were also upregulated in the EKL metatranscriptome. While GSTs can catalyze diverse reactions, when added to an existing phylogeny [63], these sequences were identified as encoding stereospecific β-etherases (*ligE*, *ligF1*), Omega-class (*ligG*), or Nu-class (GST3) GST homologs (Supplementary Fig. 15). Two *hpvZ* sequences implicated in the carboxylation of hydroxypropiovanillone or hydroxypropiosyringone were expressed, and several transcripts possibly involved in dehydrodiconiferyl alcohol degradation (encoding *DAD*, *phcD*, *lsdB*) were identified in EKL and EMWL metatranscriptomes. A putative quercetin 2,3-dioxygenase (Q23D) was also upregulated on EKL (L_2_FC = 6.6, *p*_adj_ = 0.0002).

Finally, few CAZymes showed significant upregulation on lignins (Fig. 6E). Of these, two *Altererythrobacter* AA1 transcripts were classified by the laccase engineering database [64] as 3-domain CopA-type laccase-like multicopper oxidases. Two AA4 vanillyl-alcohol oxidases in the *Altererythrobacter* transcriptome were upregulated on EKL (L_2_FC = 7.0, *p*_adj_ = 0.03; L_2_FC = 6.9, *p*_adj_ = 0.00003) and EMWL (L_2_FC = 9.3, *p*_adj_ = 0.01). Two putative pectinases were upregulated on EKL (GH28: L_2_FC = 6.0, *p*_adj_ = 3.23e−08), EMWL (PL1_2: L_2_FC = 6.6, *p*_adj_ = 0.02), and DHP (PL1_2: L_2_FC = 7.9, *p*_adj_ = 0.003).

## Discussion

Thermotolerant Alphaproteobacteria and Gammaproteobacteria from a geothermal environment expressed aromatic degradation pathways for lignin-derived monoaromatic compounds and aromatic oligomers in incubation with lignin. This finding is highly relevant to understanding the ecology of carbon cycling in geothermal environments, and has substantial biotechnology implications. In this study, two *Altererythrobacter* ssp. transcriptomes expressed complete aromatic monomer and oligomer degradation pathways during incubation with lignin (Fig. 6), despite not being fully reconstructed by genomic analysis. The syringate *meta*-cleavage pathway was primary expressed on EKL, which contained about three times higher concentration of dimethoxylated S-lignin-derived substrates than G-lignin monomers. Significantly elevated expression of *ligAB* in the *Altererythrobacter* ssp. transcriptomes suggests that syringate *meta*-cleavage in these strains proceeds via 3-*O*-methylgallate. Expression of *desB* in EKL, EMWL, and DHP also suggests that some syringate degradation occurs via gallate oxidation. Strain-level characterization of *Altererythrobacter* ssp. should resolve the relative contributions of these pathways to syringate catabolism, and characterization of the thermotolerance of these enzymes may provide new targets for biological upgrading of lignin-derived compounds.

No clear extracellular lignin-depolymerization systems were expressed in the metatranscriptomes, consistent with the apparent low mineralization of intact lignins. However, transcripts of two CopA-type LCMOs and Q23D were present in the *Altererythrobacter* transcripts. Although CopA-type LCMOs are predicted to play a role in copper detoxification, several of these enzymes have shown lignin oxidation activity [65, 66]. Similarly, a Q23D was associated with lignin depolymerization in *Pseudomonas putida* KT2440 [64], but the physiological role and substrate range of these enzymes remain uncharacterized. Finally, *Altererythrobacter* expressed genes associated with the intracellular degradation of the lignin-derived oligomers, e.g., guaiacylglycerol β-guaiacyl ether and DDVA. To date, aromatic oligomer depolymerization pathways have been identified only in the widely studied sphingomonads, SYK-6, and *N. aromaticivorans* DSM 12446 [19, 20, 61, 62, 67–69]. *Altererythrobacter* ssp. may also use Nu-class GSTs to break β-O-4 bonds [62]. As in other sphingomonads, aryl *O*-demethylation may supply essential C1 metabolites in *Altererythrobacter* strains, based on the prevalence of expressed tetrahydrofolate-dependent *O*-demethylases during lignin degradation [70]. *Altererythrobacter* spp. are typically associated with oligotrophic environments such as deep-sea sediments [71] and 45 °C hot springs [72]. Our metatranscriptomic experiment provides evidence that *Altererythrobacter* spp. catabolizes lignin-derived monoaromatic compounds via syringate and protocatechuate *meta*-cleavage pathways, and lignin-derived aromatic oligomers through several pathways including GST β-etherases.

*Rubrivivax* ssp. have diverse mechanisms to generate energy and utilize carbon including vanillate *O*-demethylation and protocatechuate *meta*-cleavage. Based on genome analyses, these thermotolerant organisms have the potential for facultative photoheterotophic growth, denitrification, CO-oxidation, H_2_-metabolism, and degradation of a variety of aromatic compounds [73–76]. Vanillate *O*-demethylase (*vanA-ivaB*) and protocatechuate 4,5-dioxygenase (*ligAB*) transcripts associated with *Rubrivivax* ssp. significantly increased in samples incubated with EKL or VAN. The *Rubrivivax* VanA had an amino acid sequence identity of 74.7% and 89.5%, respectively, to the VanA Rieske-type monooxygenases of *Comamonas testosteroni* and *Curvibacter delicatus*. A putative oxidoreductase encoded in the *Rubrivivax* transcriptome had 82.9% identity to IvaB of *C. testosteroni*, *C. testosteroni* VanA *O*-demethylates vanillate and veratrate to protocatechuate and isovanillate, respectively [77].

Genes encoding aromatic catabolic pathways, including ring-cleaving dioxygenases and GSTs, are associated with genomic adaptation to oligotrophic environments [12]. These enzymes may serve to supply carbon and energy, or carry out detoxification reactions. While we assembled protocatechuate dioxygenases in both 30 and 45 °C pools, the genes encoding *ortho*- and *meta*-cleavage enzymes were, respectively, encoded in Alphaproteobacteria (caulobacteria, *Xanthobacteraceae*) and *Burkholderiales* at each temperature. Additionally, the putative catabolism of alkylated phenols was encoded in thermophilic *Chloroflexota*. This is the first report of homologs for alkylated phenol hydroxylation encoded in *Chloroflexota* genomes, although catechol *meta*-cleavage potential was previously reported for pelagic *Chloroflexota* genomes [78]. These data demonstrate that biochemical transformations may be encoded by phylogenetically distinct organisms adapted to specific temperatures. The abundant *Burkholderiales* described herein encode multiple protocatechuate cleavage pathways and diverse energy generation mechanisms, and appear to be adapted to these highly oligotrophic geothermal environments. However, our metatranscriptomics data indicate that syringate and protocatechuate *meta*-cleavage pathways are the primary mechanisms for aromatic catabolism in these thermal swamp communities.

In this study, we demonstrate that genomic reconstruction could only partially distinguish aromatic degradation pathways, which co-occurred with pathways for facultative-photoautotrophy, halogen respiration, and arsenate detoxification in refined *Burkholderiales* (*Rubrivivax* sp.) and *Chloroflexota* (*Roseiflexaceae*) genomes. However, the targeted combination of metatranscriptomics and metabolite analysis indicated that significantly elevated expression of *Rubrivivax* sp. and sphingomonad (*Altererythrobacter* sp., *Novosphingobium* sp.) aromatic degradation pathways were associated with removal of lignin-derived substrates at 45 °C. These results supported our hypothesis that thermal swamp communities contain untapped sources of thermotolerant enzymes for lignin valorization, including copper oxidases, GSTs, and Q23Ds. The apparent preference for G- and S-lignin substrates between strains in families *Sphingomonadaceae* and *Comamonadaceae*, respectively, has implications for the use of these organisms in upgrading lignin-derived compounds. Additional approaches to characterizing hot-spring microorganisms, including isolation of identified strains, and characterization of putative lignin-depolymerization enzymes, will be critical to improve our understanding of the contribution of thermotolerant bacteria to degradation of lignin-derived aromatic compounds.

## Supplementary information

Supplemental Figures

Supplemental Methods

Data Set 1

Data Set 2

Data Set 3

Data Set 4

Data Set 5

## Data Availability

Sequence accessions are provided in Supplementary Data 2 and as part of NCBI BioProject PRJNA564648 (SRR10095339–SRR10095374). Refined genome information and NCBI accessions are provided in Supplementary Data 3.

## References

[1] Gonsior M, Hertkorn N, Hinman N, Dvorski SE-M, Harir M, Cooper WJ (2018). Yellowstone Hot Springs are organic chemodiversity hot spots. Sci Rep.

[2] Channing A, Edwards D, Sturtevant S (2004). A geothermally influenced wetland containing unconsolidated geochemical sediments. Can J Earth Sci.

[3] Channing A (2018). A review of active hot-spring analogues of Rhynie: environments, habitats and ecosystems. Philos Trans R Soc Lond B Biol Sci.

[4] Owen RB, Renaut RW, Jones B (2008). Geothermal diatoms: a comparative study of floras in hot spring systems of Iceland, New Zealand, and Kenya. Hydrobiologia..

[5] Wächtershäuser G (2006). From volcanic origins of chemoautotrophic life to Bacteria, Archaea and Eukarya. Philos Trans R Soc Lond B Biol Sci.

[6] Eloe-Fadrosh EA, Paez-Espino D, Jarett J, Dunfield PF, Hedlund BP, Dekas AE (2016). Global metagenomic survey reveals a new bacterial candidate phylum in geothermal springs. Nat Commun.

[7] Kits KD, Sedlacek CJ, Lebedeva EV, Han P, Bulaev A, Pjevac P (2017). Kinetic analysis of a complete nitrifier reveals an oligotrophic lifestyle. Nature..

[8] Costa E, Pérez J, Kreft J-U (2006). Why is metabolic labour divided in nitrification?. Trends Microbiol.

[9] Palatinszky M, Herbold C, Jehmlich N, Pogoda M, Han P, Bergen MV (2015). Cyanate as an energy source for nitrifiers. Nature..

[10] Parshina SN, Kijlstra S, Henstra AM, Sipma J, Plugge CM, Stams AJM (2005). Carbon monoxide conversion by thermophilic sulfate-reducing bacteria in pure culture and in co-culture with *Carboxydothermus hydrogenoformans*. Appl Microbiol Biotechnol.

[11] Brady AL, Sharp CE, Grasby SE, Dunfield PF (2015). Anaerobic carboxydotrophic bacteria in geothermal springs identified using stable isotope probing. Front Microbiol.

[12] Lauro FM, McDougald D, Thomas T, Williams TJ, Egan S, Rice S (2009). The genomic basis of trophic strategy in marine bacteria. Proc Natl Acad Sci USA.

[13] Ceballos SJ, Yu C, Claypool JT, Singer SW, Simmons BA, Thelen MP (2017). Development and characterization of a thermophilic, lignin degrading microbiota. Process Biochem.

[14] Fernandes TAR, da Silveira WB, Passos FML, Zucchi TD (2014). Characterization of a thermotolerant laccase produced by *Streptomyces* sp. SB086. Ann Microbiol.

[15] Taylor CR, Hardiman EM, Ahmad M, Sainsbury PD, Norris PR, Bugg TDH (2012). Isolation of bacterial strains able to metabolize lignin from screening of environmental samples. J Appl Microbiol.

[16] Levy-Booth DJ, Fetherolf MM, Stewart GR, Liu J, Eltis LD, Mohn WW (2019). Catabolism of alkylphenols in *Rhodococcus* via a *meta*-cleavage pathway associated with genomic islands. Front Microbiol.

[17] Perez JM, Kontur WS, Alherech M, Coplien J, Karlen SD, Stahl SS (2019). Funneling aromatic products of chemically depolymerized lignin into 2-pyrone-4-6-dicarboxylic acid with *Novosphingobium aromaticivorans*. Green Chem.

[18] Beckham GT, Johnson CW, Karp EM, Salvachúa D, Vardon DR (2016). Opportunities and challenges in biological lignin valorization. Curr Opin Biotechnol.

[19] Masai E, Kamimura N, Kasai D, Oguchi A, Ankai A, Fukui S (2012). Complete genome sequence of *Sphingobium* sp. strain SYK-6, a degrader of lignin-derived biaryls and monoaryls. J Bacteriol.

[20] Cecil JH, Garcia DC, Giannone RJ, Michener JK (2018). Rapid, parallel identification of catabolism pathways of lignin-derived aromatic compounds in *Novosphingobium aromaticivorans*. Appl Environ Microbiol.

[21] Tasaki M, Kamagata Y, Nakamura K, Mikami E (1992). Utilization of methoxylated benzoates and formation of intermediates by *Desulfotomaculum thermobenzoicum* in the presence or absence of sulfate. Arch Microbiol.

[22] Kato S, Chino K, Kamimura N, Masai E, Yumoto I, Kamagata Y (2015). Methanogenic degradation of lignin-derived monoaromatic compounds by microbial enrichments from rice paddy field soil. Sci Rep.

[23] Daniel SL, Keith ES, Yang H, Lin Y-S, Drake HL (1991). Utilization of methoxylated aromatic compounds by the acetogen *Clostridium thermoaceticum*: Expression and specificity of the co-dependent *O*-demethylating activity. Biochem Biophys Res Commun.

[24] Daniel SL, Wu Z, Drake HL (1988). Growth of thermophilic acetogenic bacteria on methoxylated aromatic acids. FEMS Microbiol Lett.

[25] Kasai D, Masai E, Miyauchi K, Katayama Y, Fukuda M (2005). Characterization of the gallate dioxygenase gene: three distinct ring cleavage dioxygenases are involved in syringate degradation by *Sphingomonas paucimobilis* SYK-6. J Bacteriol.

[26] Kasai D, Masai E, Katayama Y, Fukuda M (2007). Degradation of 3-*O*-methylgallate in *Sphingomonas paucimobilis* SYK-6 by pathways involving protocatechuate 4,5-dioxygenase. FEMS Microbiol Lett.

[27] Li H, Yang Q, Li J, Gao H, Li P, Zhou H (2015). The impact of temperature on microbial diversity and AOA activity in the Tengchong Geothermal Field, China. Sci Rep.

[28] Nottingham AT, Fierer N, Turner BL, Whitaker J, Ostle NJ, McNamara NP (2018). Microbes follow Humboldt: temperature drives plant and soil microbial diversity patterns from the Amazon to the Andes. Ecology..

[29] Sharp CE, Brady AL, Sharp GH, Grasby SE, Stott MB, Dunfield PF (2014). Humboldt’s spa: microbial diversity is controlled by temperature in geothermal environments. ISME J.

[30] Heron J, Sheffield C (2016). First Canadian record of the water mite *Thermacarus nevadensis* Marshall, 1928 (*Arachnida*: *Acariformes*: *Hydrachnidiae*: *Thermacaridae*) from hot springs in British Columbia. Biodivers Data J..

[31] Grasby SE, Hutcheon I, Krouse HR (2000). The influence of water–rock interaction on the chemistry of thermal springs in western Canada. Appl Geochem.

[32] Grasby SE, Ferguson G, Brady A, Sharp C, Dunfield P, McMechan M (2016). Deep groundwater circulation and associated methane leakage in the northern Canadian Rocky Mountains. Appl Geochem.

[33] Bauchop T, Elsden SR (1960). The growth of micro-organisms in relation to their energy supply. Microbiol.

[34] Elder R. Cloning Techniques. BioScience. 1983;33:721–2. https://www.jstor.org/stable/1309366.

[35] Wilhelm RC, Singh R, Eltis LD, Mohn WW (2018). Bacterial contributions to delignification and lignocellulose degradation in forest soils with metagenomic and quantitative stable isotope probing. ISME J.

[36] Griffiths RI, Whiteley AS, O’Donnell AG, Bailey MJ (2000). Rapid method for coextraction of DNA and RNA from natural environments for analysis of ribosomal DNA- and rRNA-based microbial community composition. Appl Environ Microbiol.

[37] Luo XZ, Stevens SE (1997). Isolation of full-length RNA from a thermophilic cyanobacterium. BioTechniques..

[38] Shieh T-M, Chen C-Y, Hsueh C, Yu C-C, Chen C-C, Wang T-H (2018). Application of ribonucleoside vanadyl complex (RVC) for developing a multifunctional tissue preservative solution. PLoS ONE.

[39] Das L, Li M, Stevens J, Li W, Pu Y, Ragauskas AJ (2018). Characterization and catalytic transfer hydrogenolysis of deep eutectic solvent extracted sorghum lignin to phenolic compounds. ACS Sustain Chem Eng.

[40] Balakshin MY, Capanema EA, Santos RB, Chang H, Jameel H (2016). Structural analysis of hardwood native lignins by quantitative ^13^C NMR spectroscopy. Holzforschung..

[41] Holtman KM, Chang H, Jameel H, Kadla JF (2006). Quantitative ^13^C NMR characterization of milled wood lignins isolated by different milling techniques. J Wood Chem Technol.

[42] Bolger AM, Lohse M, Usadel B (2014). Trimmomatic: a flexible trimmer for Illumina sequence data. Bioinformatics..

[43] Li D, Liu C-M, Luo R, Sadakane K, Lam T-W (2015). MEGAHIT: an ultra-fast single-node solution for large and complex metagenomics assembly via succinct de Bruijn graph. Bioinformatics..

[44] Hyatt D, Chen G-L, LoCascio PF, Land ML, Larimer FW, Hauser LJ (2010). Prodigal: prokaryotic gene recognition and translation initiation site identification. BMC Bioinform.

[45] Menzel P, Ng KL, Krogh A (2016). Fast and sensitive taxonomic classification for metagenomics with Kaiju. Nat Commun.

[46] Kang D, Li F, Kirton ES, Thomas A, Egan RS, An H (2019). MetaBAT 2: an adaptive binning algorithm for robust and efficient genome reconstruction from metagenome assemblies. PeerJ.

[47] Parks DH, Imelfort M, Skennerton CT, Hugenholtz P, Tyson GW (2015). CheckM: assessing the quality of microbial genomes recovered from isolates, single cells, and metagenomes. Genome Res.

[48] Parks DH, Chuvochina M, Waite DW, Rinke C, Skarshewski A, Chaumeil P-A (2018). A standardized bacterial taxonomy based on genome phylogeny substantially revises the tree of life. Nat Biotechnol.

[49] Haft DH, Loftus BJ, Richardson DL, Yang F, Eisen JA, Paulsen IT (2001). TIGRFAMs: a protein family resource for the functional identification of proteins. Nucleic Acids Res.

[50] El-Gebali S, Mistry J, Bateman A, Eddy SR, Luciani A, Potter SC (2019). The Pfam protein families database in 2019. Nucleic Acids Res.

[51] Anantharaman K, Brown CT, Hug LA, Sharon I, Castelle CJ, Probst AJ (2016). Thousands of microbial genomes shed light on interconnected biogeochemical processes in an aquifer system. Nat Commun.

[52] Aramaki T, Blanc-Mathieu R, Endo H, Ohkubo K, Kanehisa M, Goto S (2019). KofamKOALA: KEGG ortholog assignment based on profile HMM and adaptive score threshold. Bioinformatics.

[53] Tourna M, Stieglmeier M, Spang A, Könneke M, Schintlmeister A, Urich T (2011). *Nitrososphaera viennensis*, an ammonia oxidizing archaeon from soil. PNAS..

[54] Zhang H, Yohe T, Huang L, Entwistle S, Wu P, Yang Z (2018). dbCAN2: a meta server for automated carbohydrate-active enzyme annotation. Nucleic Acids Res.

[55] R Core Team. R: a language and environment for statistical computing. Vienna: R Foundation for Statistical Computing; 2018. https://www.R-project.org.

[56] Love MI, Huber W, Anders S (2014). Moderated estimation of fold change and dispersion for RNA-seq data with DESeq2. Genome Biol.

[57] Takeda M, Kamagata Y, Ghiorse WC, Hanada S, Koizumi J (2002). *Caldimonas manganoxidans* gen. nov., sp. nov., a poly(3-hydroxybutyrate)-degrading, manganese-oxidizing thermophile. Int J Syst Evol Microbiol.

[58] Lombard V, Golaconda Ramulu H, Drula E, Coutinho PM, Henrissat B (2014). The carbohydrate-active enzymes database (CAZy) in 2013. Nucleic Acids Res.

[59] Suzek BE, Wang Y, Huang H, McGarvey PB, Wu CH (2015). UniRef clusters: a comprehensive and scalable alternative for improving sequence similarity searches. Bioinformatics..

[60] Pattrick CA, Webb JP, Green J, Chaudhuri RR, Collins MO, Kelly DJ (2019). Proteomic profiling, transcription factor modeling, and genomics of evolved tolerant strains elucidate mechanisms of vanillin toxicity in *Escherichia coli*. mSystems.

[61] Yoshikata T, Suzuki K, Kamimura N, Namiki M, Hishiyama S, Araki T (2014). Three-component *O*-demethylase system essential for catabolism of a lignin-derived biphenyl compound in *Sphingobium* sp. strain SYK-6. Appl Environ Microbiol.

[62] Kontur WS, Bingman CA, Olmsted CN, Wassarman DR, Ulbrich A, Gall DL (2018). *Novosphingobium aromaticivorans* uses a Nu-class glutathione S-transferase as a glutathione lyase in breaking the β-aryl ether bond of lignin. J Biol Chem.

[63] Kontur WS, Olmsted CN, Yusko LM, Niles AV, Walters KA, Beebe ET (2018). A heterodimeric glutathione S-transferase that stereospecifically breaks lignin’s β(R)-aryl ether bond reveals the diversity of bacterial β-etherases. J Biol Chem.

[64] Salvachúa D, Werner AZ, Pardo I, Michalska M, Black BA, Donohoe BS (2020). Outer membrane vesicles catabolize lignin-derived aromatic compounds in *Pseudomonas putida* KT2440. PNAS..

[65] Granja-Travez RS, Bugg TDH (2018). Characterization of multicopper oxidase CopA from *Pseudomonas putida* KT2440 and *Pseudomonas fluorescens* Pf-5: Involvement in bacterial lignin oxidation. Arch Biochem Biophys.

[66] Strachan CR, Singh R, VanInsberghe D, Ievdokymenko K, Budwill K, Mohn WW (2014). Metagenomic scaffolds enable combinatorial lignin transformation. PNAS..

[67] Masai E, Sasaki M, Minakawa Y, Abe T, Sonoki T, Miyauchi K (2004). A novel tetrahydrofolate-dependent *O-*demethylase gene is essential for growth of *Sphingomonas paucimobilis* SYK-6 with syringate. J Bacteriol.

[68] Abe T, Masai E, Miyauchi K, Katayama Y, Fukuda M (2005). A tetrahydrofolate-dependent *O-*demethylase, LigM, is crucial for catabolism of vanillate and syringate in *Sphingomonas paucimobilis* SYK-6. J Bacteriol.

[69] Sonoki T, Obi T, Kubota S, Higashi M, Masai E, Katayama Y (2000). Coexistence of two different *O* demethylation systems in lignin metabolism by *Sphingomonas paucimobilis* SYK-6: cloning and sequencing of the lignin biphenyl-specific *O*-demethylase (LigX) gene. Appl Environ Microbiol.

[70] Sonoki T, Masai E, Sato K, Kajita S, Katayama Y (2009). Methoxyl groups of lignin are essential carbon donors in C1 metabolism of *Sphingobium* sp. SYK-6. J Basic Microbiol.

[71] Maeda AH, Nishi S, Ishii S, Shimane Y, Kobayashi H, Ichikawa J (2018). Complete genome sequence of *Altererythrobacter* sp. strain B11, an aromatic monomer-degrading bacterium, isolated from deep-sea sediment under the seabed off Kashima, Japan. Genome Announc.

[72] Yuan C-G, Chen X, Jiang Z, Chen W, Liu L, Xian W-D (2017). *Altererythrobacter lauratis* sp. nov. and *Altererythrobacter palmitatis* sp. nov., isolated from a Tibetan hot spring. Antonie Van Leeuwenhoek.

[73] Nagashima S, Kamimura A, Shimizu T, Nakamura-Isaki S, Aono E, Sakamoto K (2012). Complete genome sequence of phototrophic betaproteobacterium *Rubrivivax gelatinosus* IL144. J Bacteriol.

[74] Sheu S-Y, Li Z-H, Young C-C, Chen W-M (2020). *Rubrivivax albus* sp. nov., isolated from a freshwater pond. Int J Syst Evol Microbiol.

[75] Ramana ChV, Sasikala CH, Arunasri K, Anil Kumar P, Srinivas TNR, Shivaji S (2006). *Rubrivivax benzoatilyticus* sp. nov., an aromatic, hydrocarbon-degrading purple betaproteobacterium. Int J Syst Evol Microbiol.

[76] Wawrousek K, Noble S, Korlach J, Chen J, Eckert C, Yu J (2014). Genome annotation provides insight into carbon monoxide and hydrogen metabolism in *Rubrivivax gelatinosus*. PLoS ONE.

[77] Providenti MA, O’Brien JM, Ruff J, Cook AM, Lambert IB (2006). Metabolism of isovanillate, vanillate, and veratrate by *Comamonas testosteroni* strain BR6020. J Bacteriol.

[78] Colatriano D, Tran PQ, Guéguen C, Williams WJ, Lovejoy C, Walsh DA (2018). Genomic evidence for the degradation of terrestrial organic matter by pelagic Arctic Ocean Chloroflexi bacteria. Commun Biol..

