# Supplementary material for: Genomics and metatranscriptomics of biogeochemical cycling and degradation of lignin-derived aromatic compounds in thermal swamp sediment
Source: ISME J. 2020 Nov 2;15(3):879–93. doi: 10.1038/s41396-020-00820-x (PMC8027834; doi:10.1038/s41396-020-00820-x)
Supplement: Supplementary file 1 — Supplemental Figures [file 41396_2020_820_MOESM1_ESM.pdf]

# Genomics and metatranscriptomics of biogeochemical cycling and degradation of lignin-derived aromatic compounds in thermal swamp sediment

David J. Levy-Booth[1], Ameena Hashimi[1], Raphael Roccor[1], Liyang Liu[2], Scott Renneckar[2] , Lindsay D. Eltis[1], William W. Mohn[1*]

1. Department of Microbiology and Immunology, The University of British Columbia, Vancouver, Canada
2. Department of Wood Science, The University of British Columbia, Vancouver, Canada

Running title: Aromatic catabolism in a thermal metatranscriptome

[*]To whom correspondence should be addressed: William W. Mohn, Department of Microbiology and Immunology, Life Sciences Institute, The University of British Columbia, 2350 Health Sciences Mall, Vancouver, BC, V6T 1Z3, Canada. Tel.: +1- 604–822–4285; Fax: +1-604-822-6041; E-mail: wmohn@mail.ubc.ca

**Supplementary Figures**

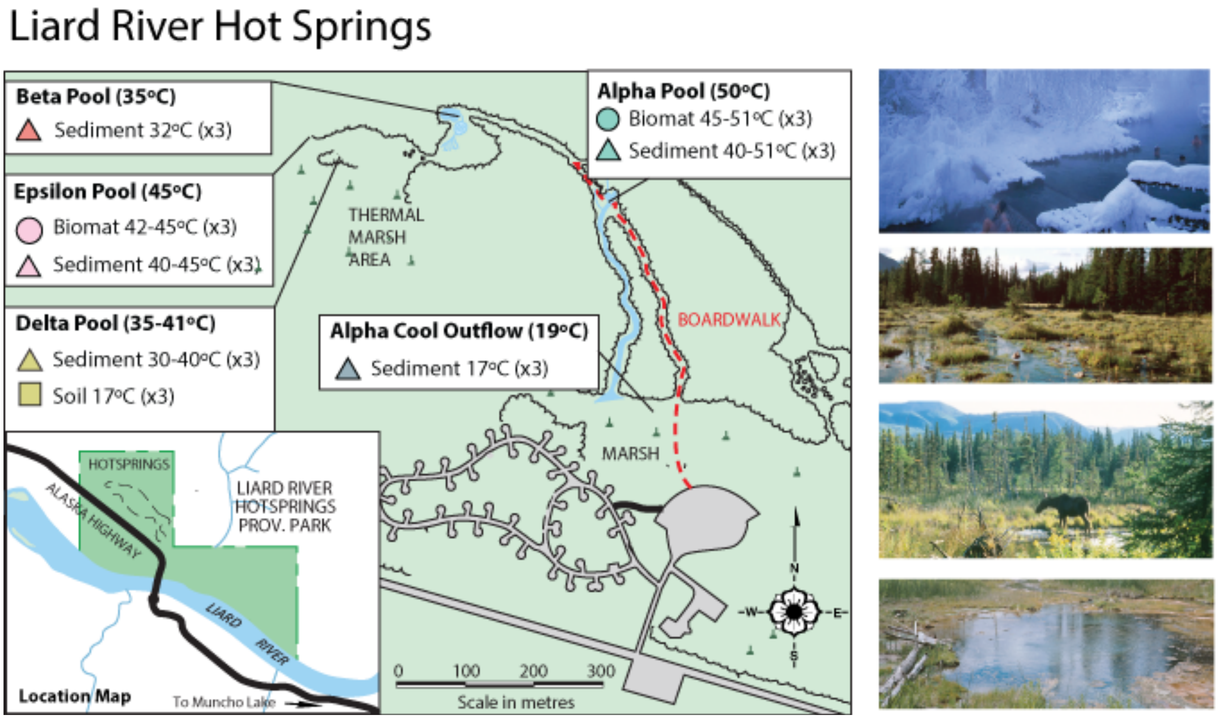


Supplementary Figure 1. Map of the Liard River Hot Springs, including sampling sites used in this study. Photos by D. Levy-Booth and Parks BC.

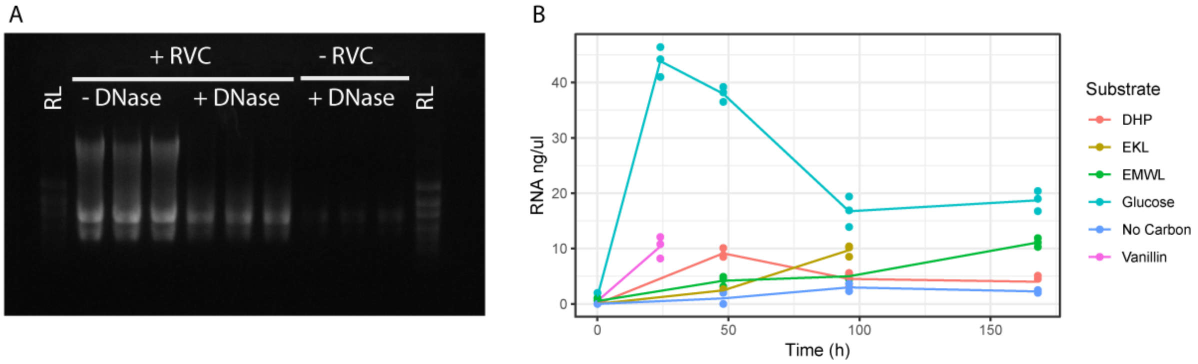


Supplementary Figure 2. RNA Extraction. A) RNA extracted from Epsilon incubated with 0.1% (v/w) glucose for 24 hr amended with 20 mM ribonucleoside vanadyl complex (RVC) before and after DNase treatment. RVC prevents the degradation of RNA during extraction. In compassion, unamended extracts are shown post-DNase treatment. RL, RNA ladder. B) RNA yield during 168 hr incubations.

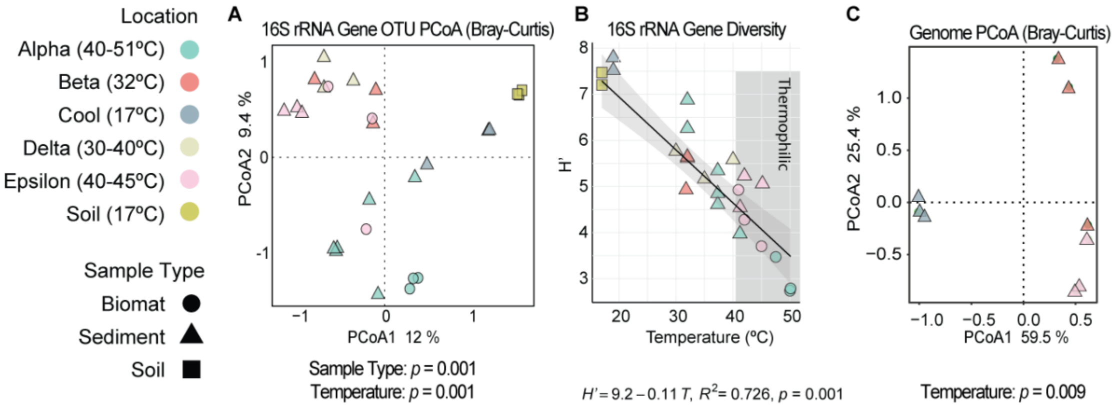


Supplementary Figure 3. Effect of temperature on microbial diversity. A) PCoA analysis of 16S rRNA OTU Bray-Curtis (BC) dissimilarly across thermal swamp complex communities. Effect of sample type (biomat, sediment, soil) and temperature on BC dissimilarity calculated with PERMANOVA. B) Linear regression showing the effect of temperature (*T*) on Shannon-Weiner diversity (H'). C) PCoA analysis of genome Bray-Curtis (BC) dissimilarly across thermal swamp complex communities. Effect of temperature on BC dissimilarity calculated with PERMANOVA.

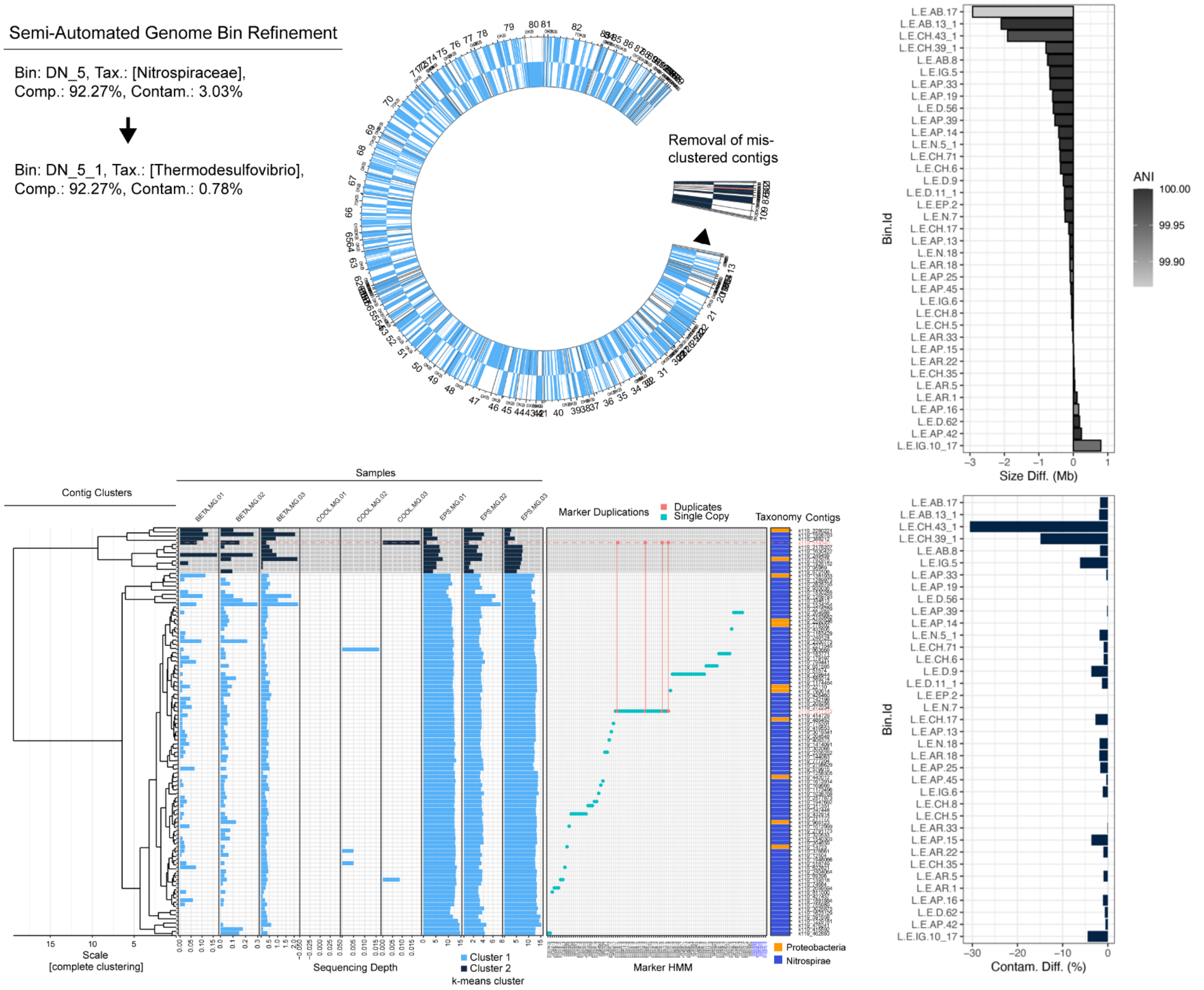


Supplementary Figure 4. Contig bin refinement methods resulted in reduced contamination and improved taxonomic classification. Contigs were clustered by depth across samples using average-linkage hierarchical clustering and k-means clustering, annotated for single-copy genes (SCGs) with CheckM, and assessed for consensus taxonomy with Kaiju classification. Contigs clustering separately from bulk of binned contigs and containing duplicate SCGs were removed from the final assembly. Right panel top: Net change in genome size for 37 refined genomes, shaded by ANI between initial and final genome. Right panel bottom: Change in contamination following genome refinement.

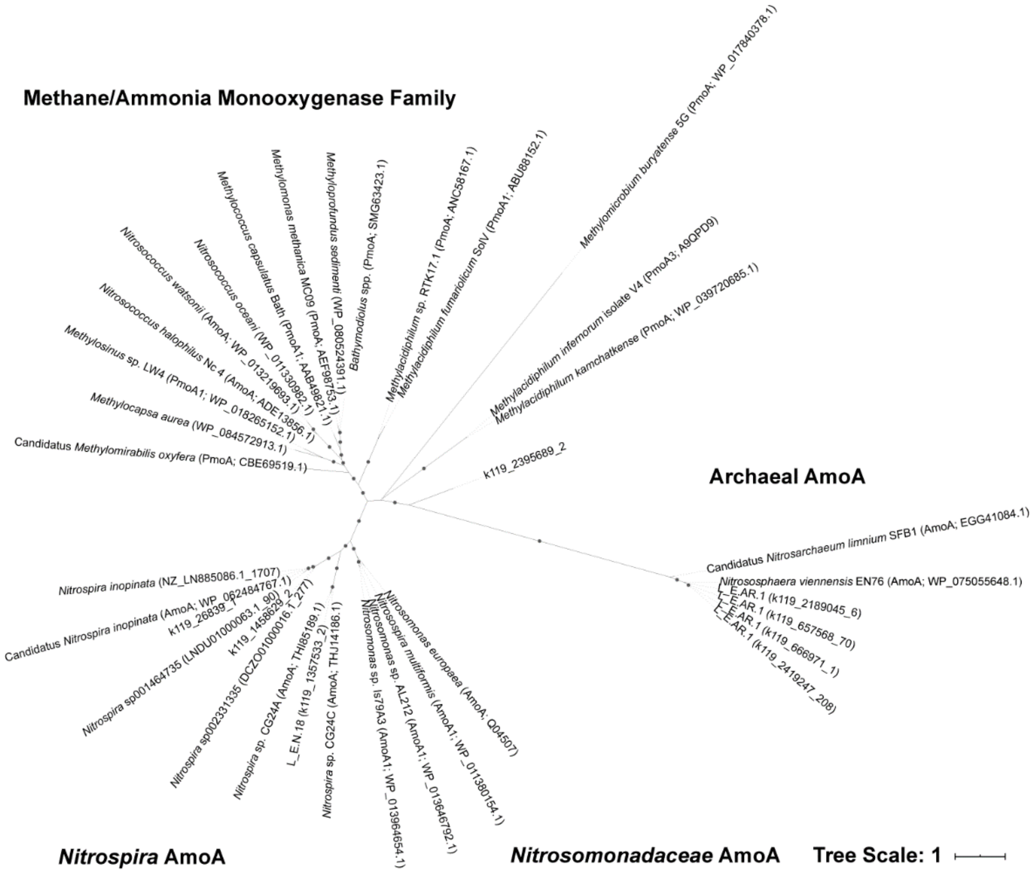


Supplemental Figure 5. Phylogeny of particulate methane monooxygenase (PmoA) and ammonia monooxygenase (AmoA) sequences based on amino acid alignment with MAFFT v7.407 (ginsi settings, 1000 maximum iterations). Alignment was trimmed using trimal (1.2rev59) with a gap threshold of 20%. Phylogeny was calculated using FastTree 2.1.10 using default settings. Bootstrap support of >75% is shown (black circles).

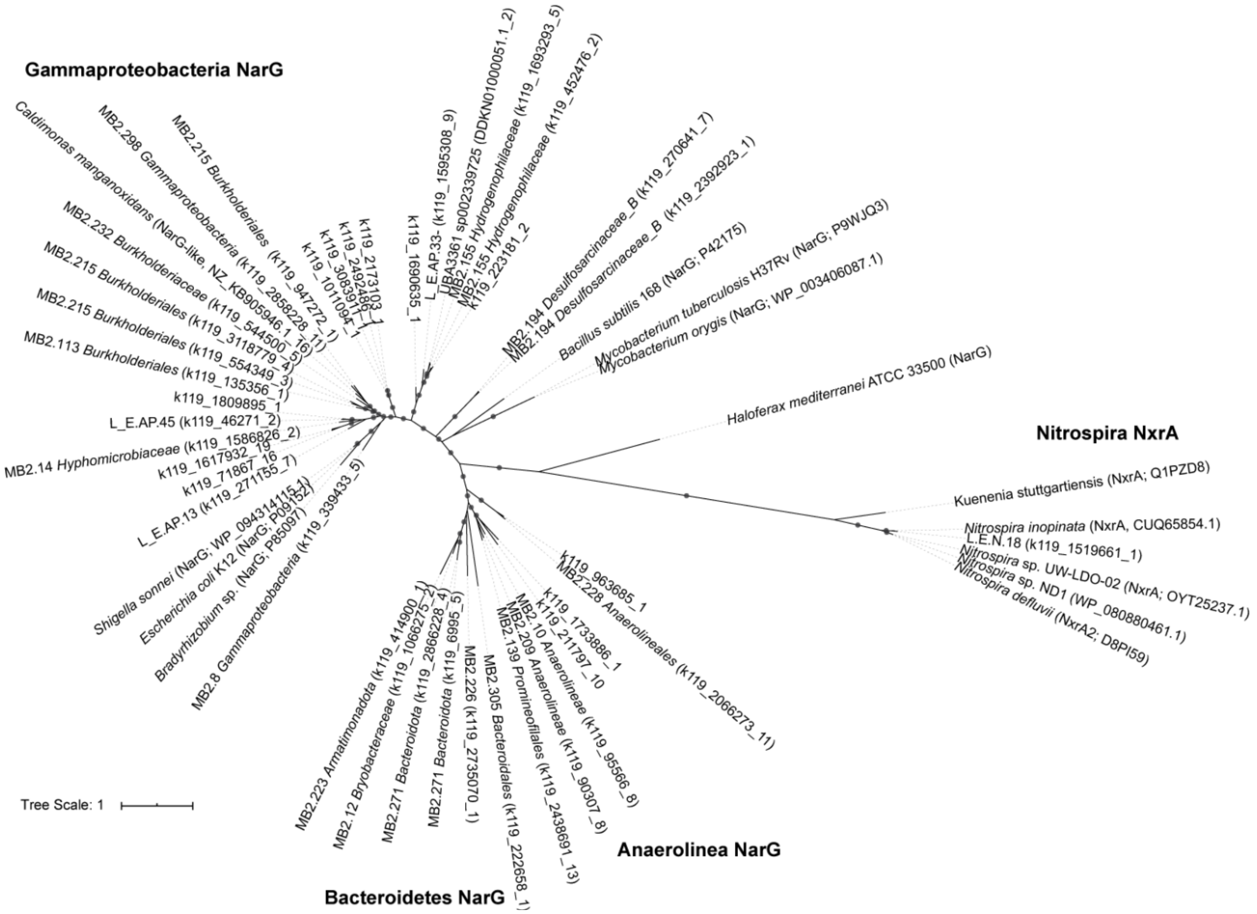

Supplemental Figure 6. Phylogeny of Nitrite oxidoreductase (NxrA) and nitrate reductase (NarG) alpha subunits based on amino acid alignment with MAFFT v7.407 (ginsi settings, 1000 maximum iterations). Alignment was trimmed using trimal (1.2rev59) with a gap threshold of 20%. Phylogeny was calculated using FastTree 2.1.10 using default settings. Bootstrap support of >75% is shown (black circles).

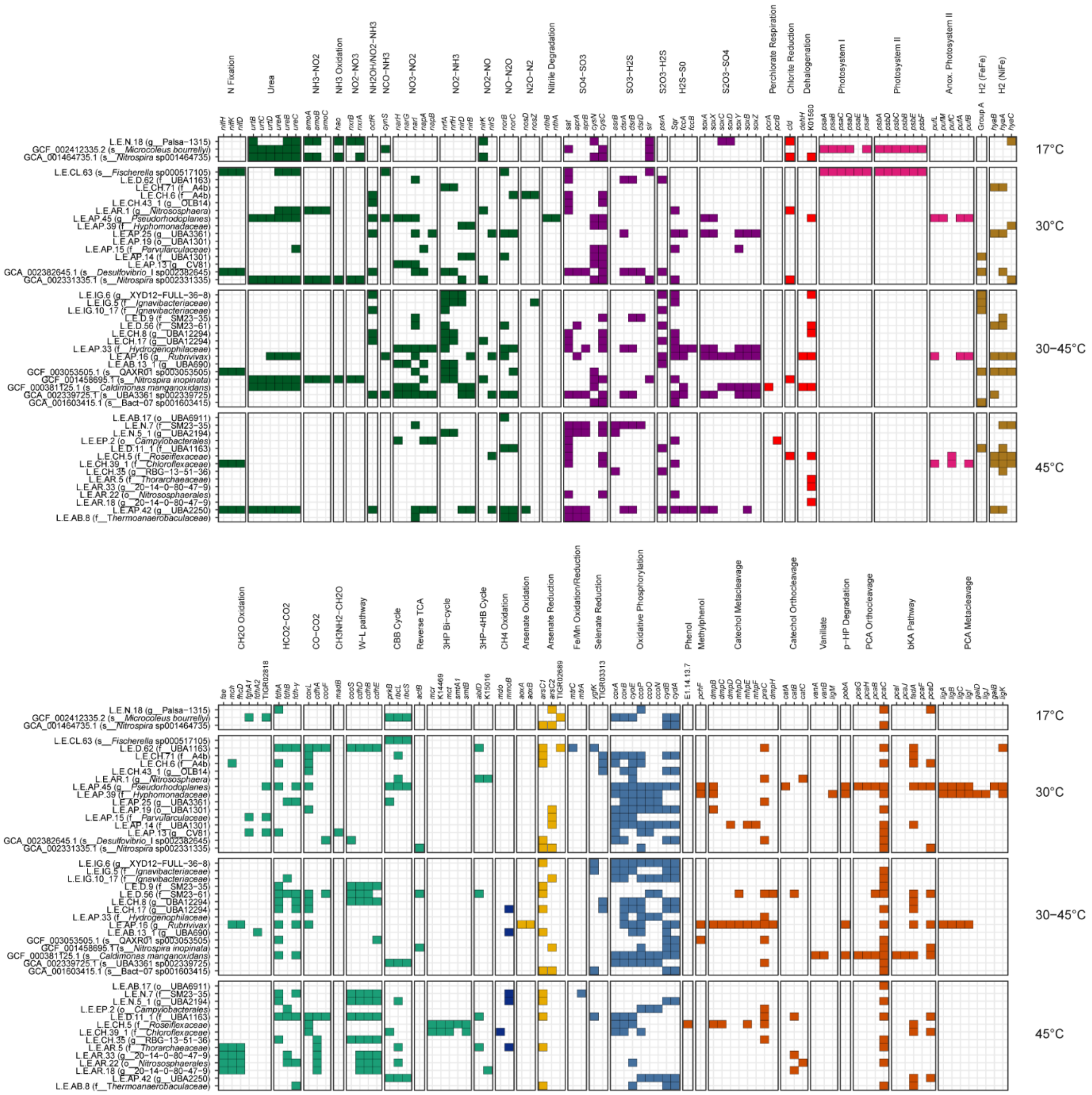


Supplemental Figure 7. HMM-based annotation of metabolic pathway genes in draft genomes.

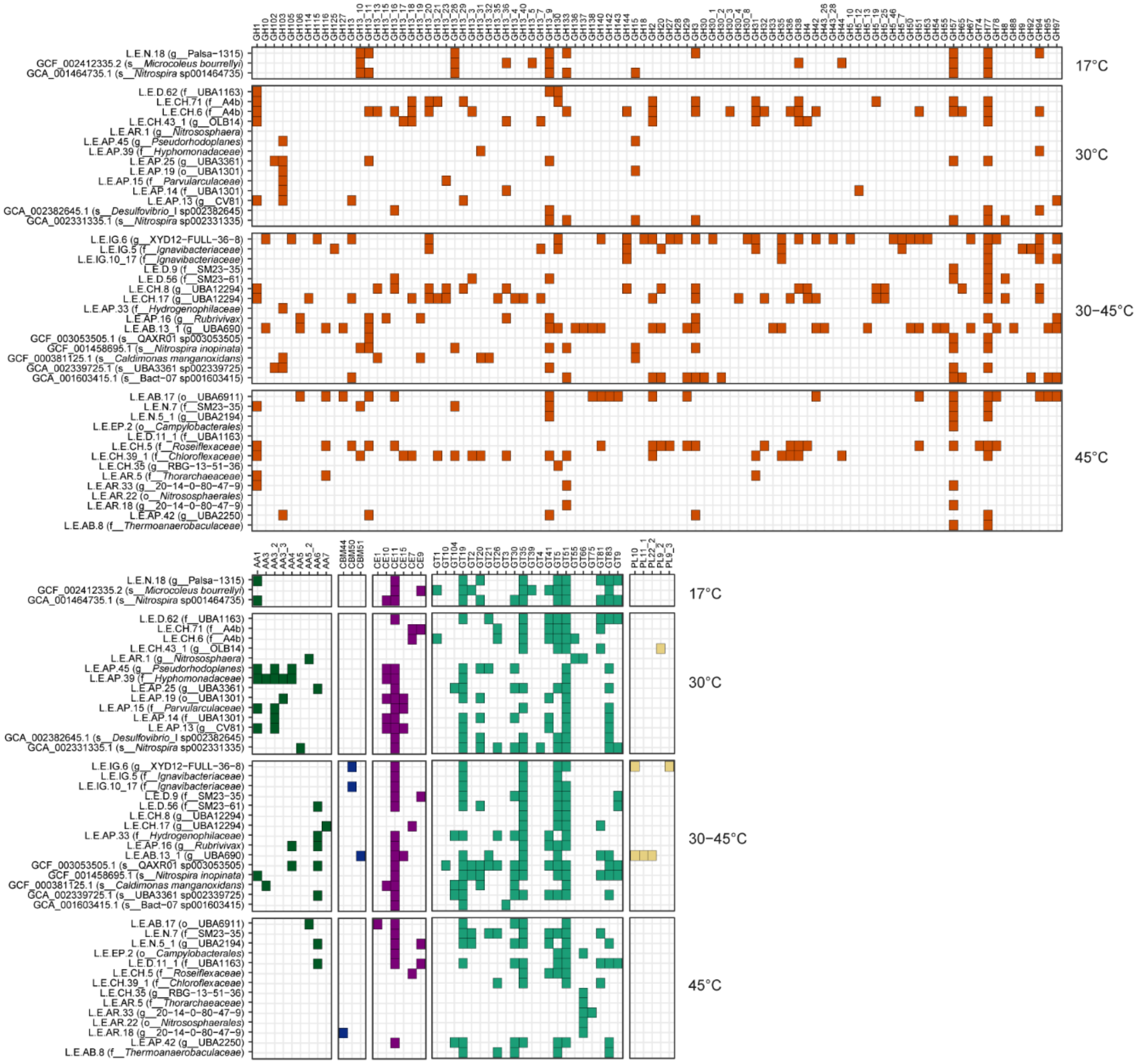

Supplemental Figure 8. HMM-based annotation of carbohydrate-active enzymes (CAZymes) in draft genomes using dbCAN2 and the CAZy database.

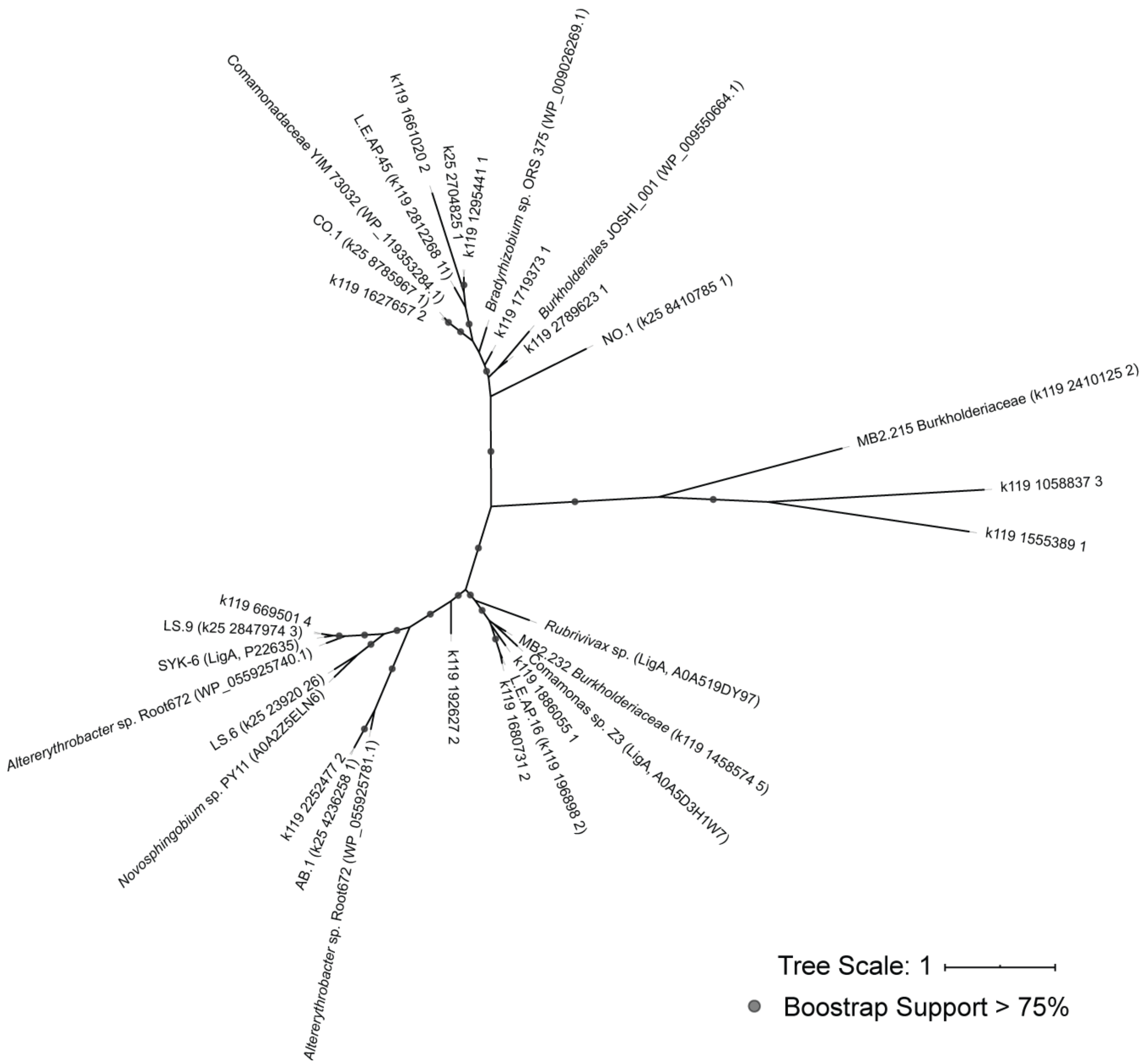


Supplemental Figure 9. Phylogenetic placement of protocatechuate 4,5-dioxygenase alpha subunit gene and transcript amino acid sequences from metagenomics and metatranscriptome assemblies were aligned with reference sequences using MAFFT 7.407 using ginsi settings with a maximum of 1000 iterations. Phylogeny was calculated using FastTree.

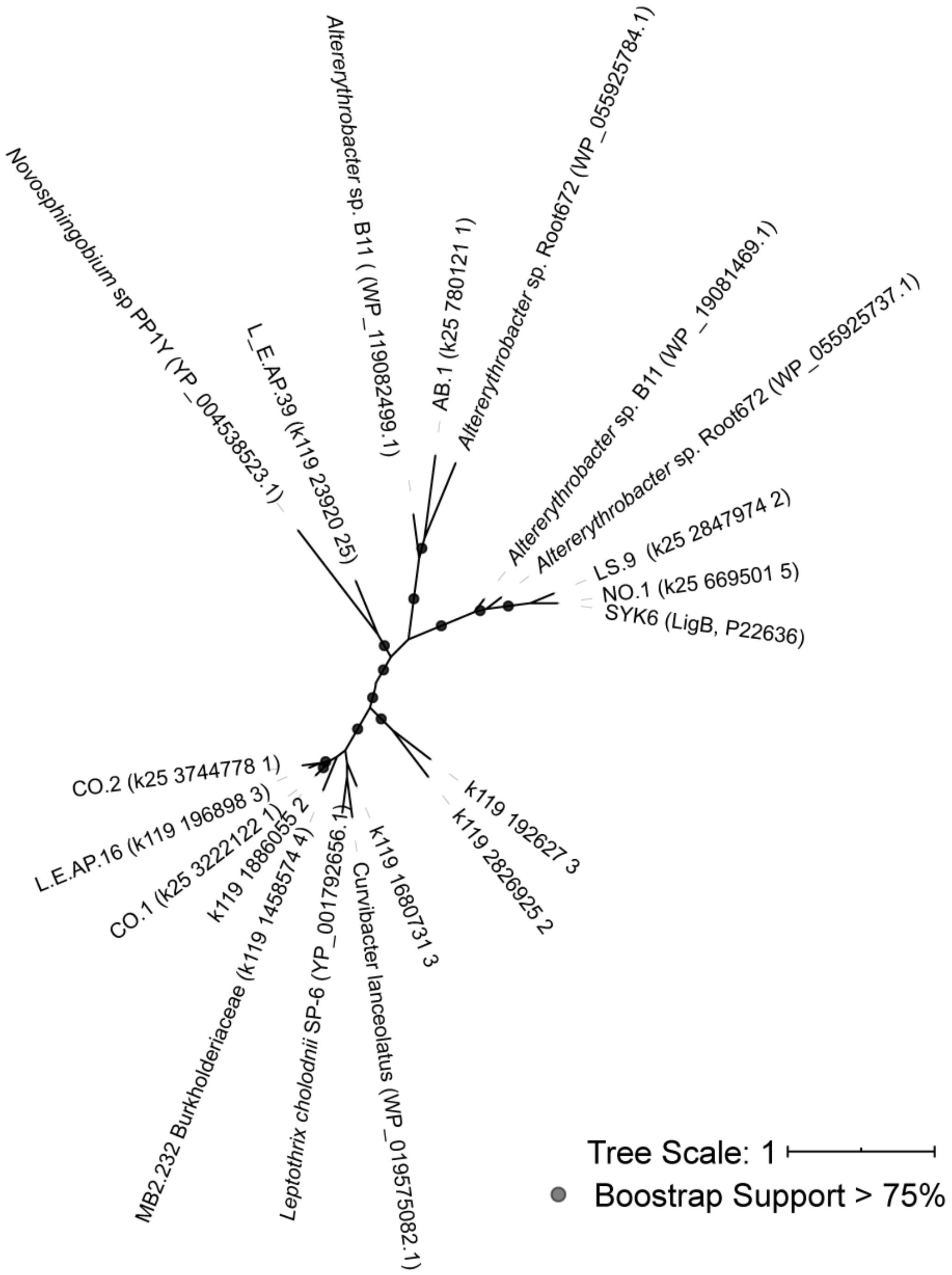


Supplemental Figure 10. Phylogenetic placement of protocatechuate 4,5-dioxygenase beta subunit gene and transcript amino acid sequences from metagenomics and metatranscriptome assemblies were aligned with reference sequences using MAFFT 7.407 using ginsi settings with a maximum of 1000 iterations. Phylogeny was calculated using FastTree.

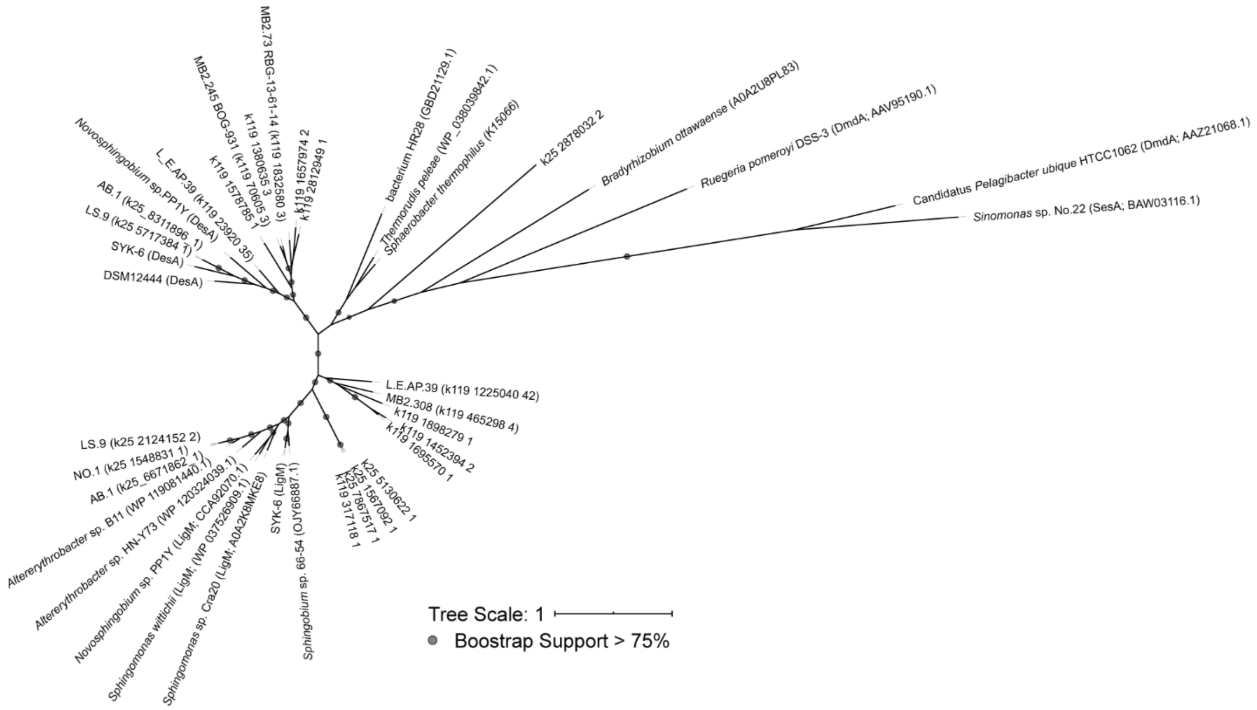

Supplemental Figure 11. Phylogenetic placement of tetrahydrofolate-dependent aromatic O-demethylase gene and transcript amino acid sequences from metagenomics and metatranscriptome assemblies were aligned with reference sequences using MAFFT 7.407 using ginsi settings with a maximum of 1000 iterations. Phylogeny was calculated using FastTree.

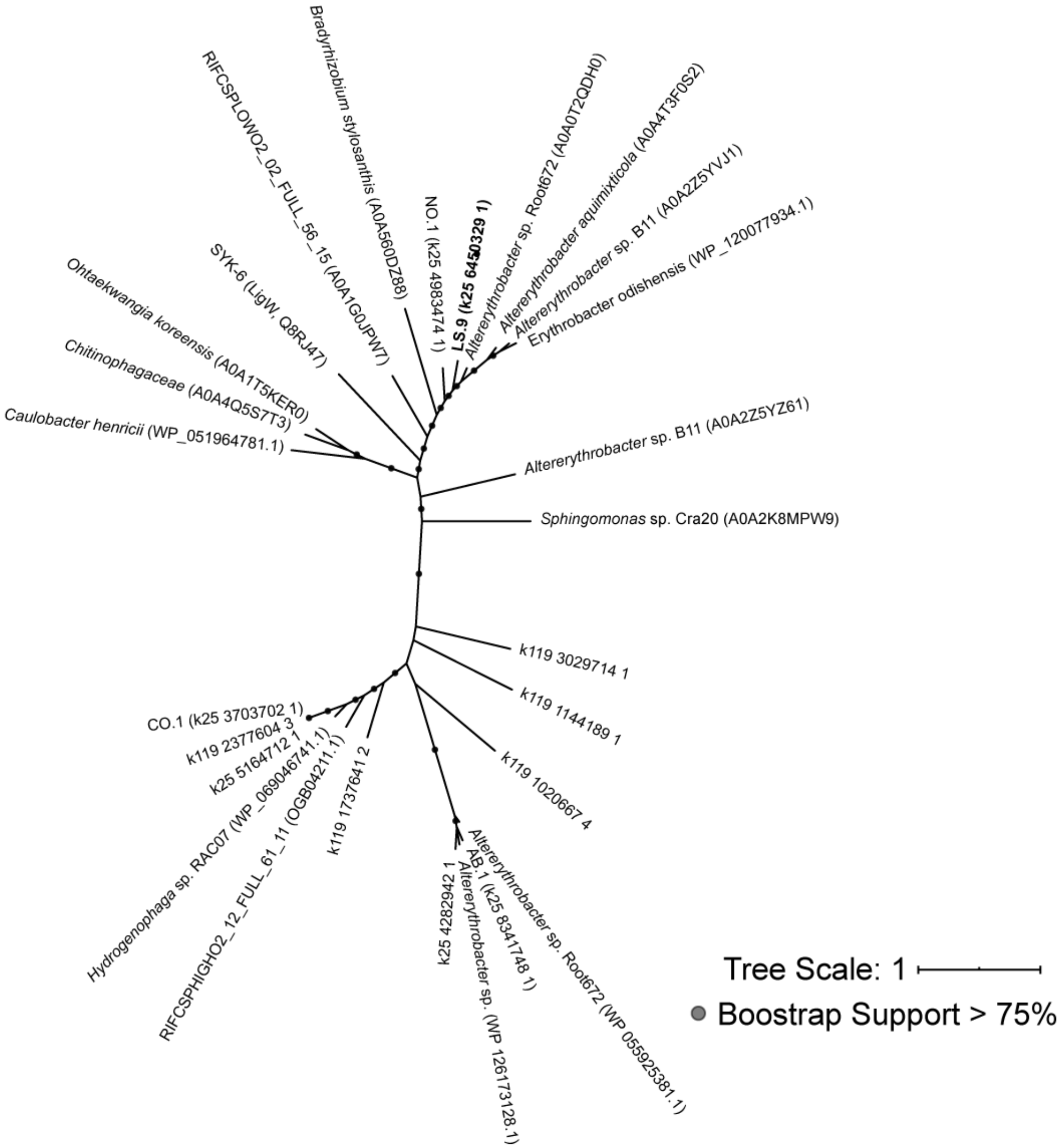


Supplemental Figure 12. Phylogenetic placement of 5-carboxyvanillate decarboxylase gene and transcript amino acid sequences from metagenomics and metatranscriptome assemblies were aligned with reference sequences using MAFFT 7.407 using ginsi settings with a maximum of 1000 iterations. Phylogeny was calculated using FastTree.

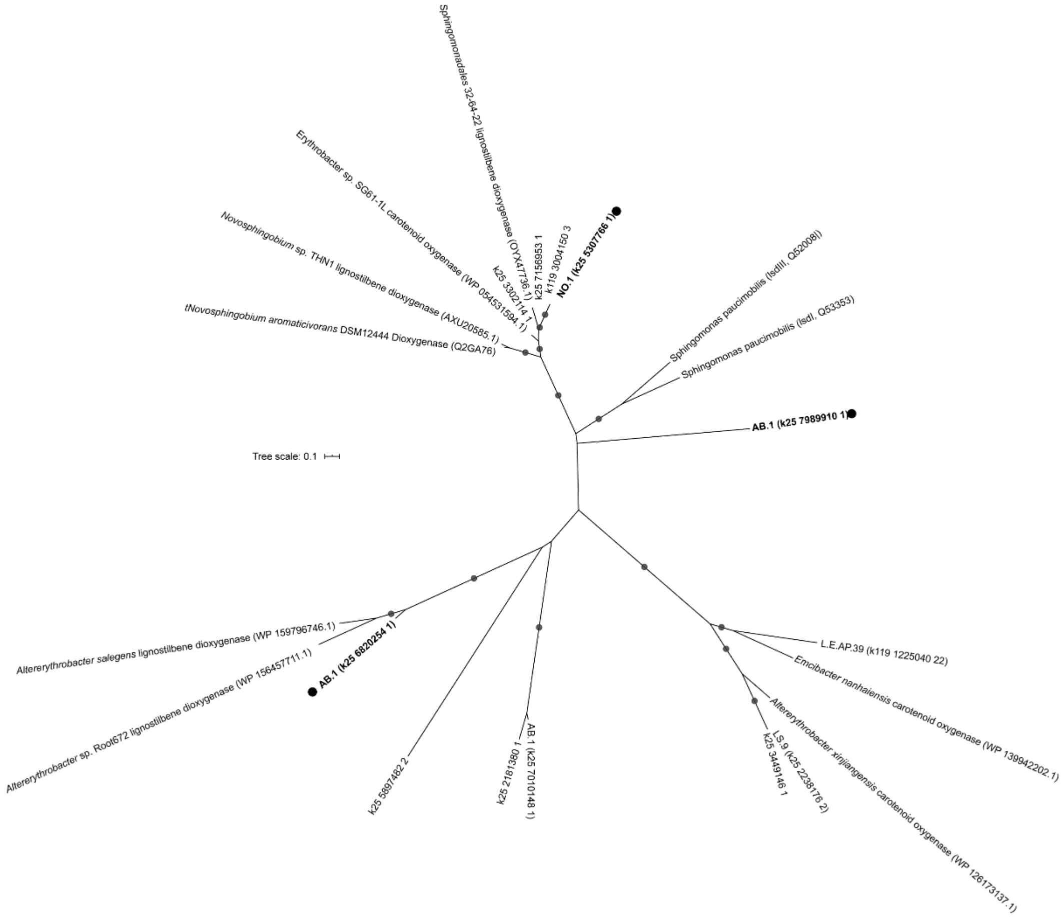


Supplementary Figure 13. Phylogenetic placement of assembled genes and transcripts with lignostilbene alpha-beta dioxygenase and other carotenoid oxygenase family proteins. Amino acid sequences from metagenomics and metatranscriptome assemblies were aligned with reference sequences using MAFFT 7.407 using ginsi settings with a maximum of 1000 iterations, and phylogeny calculated using FastTree.

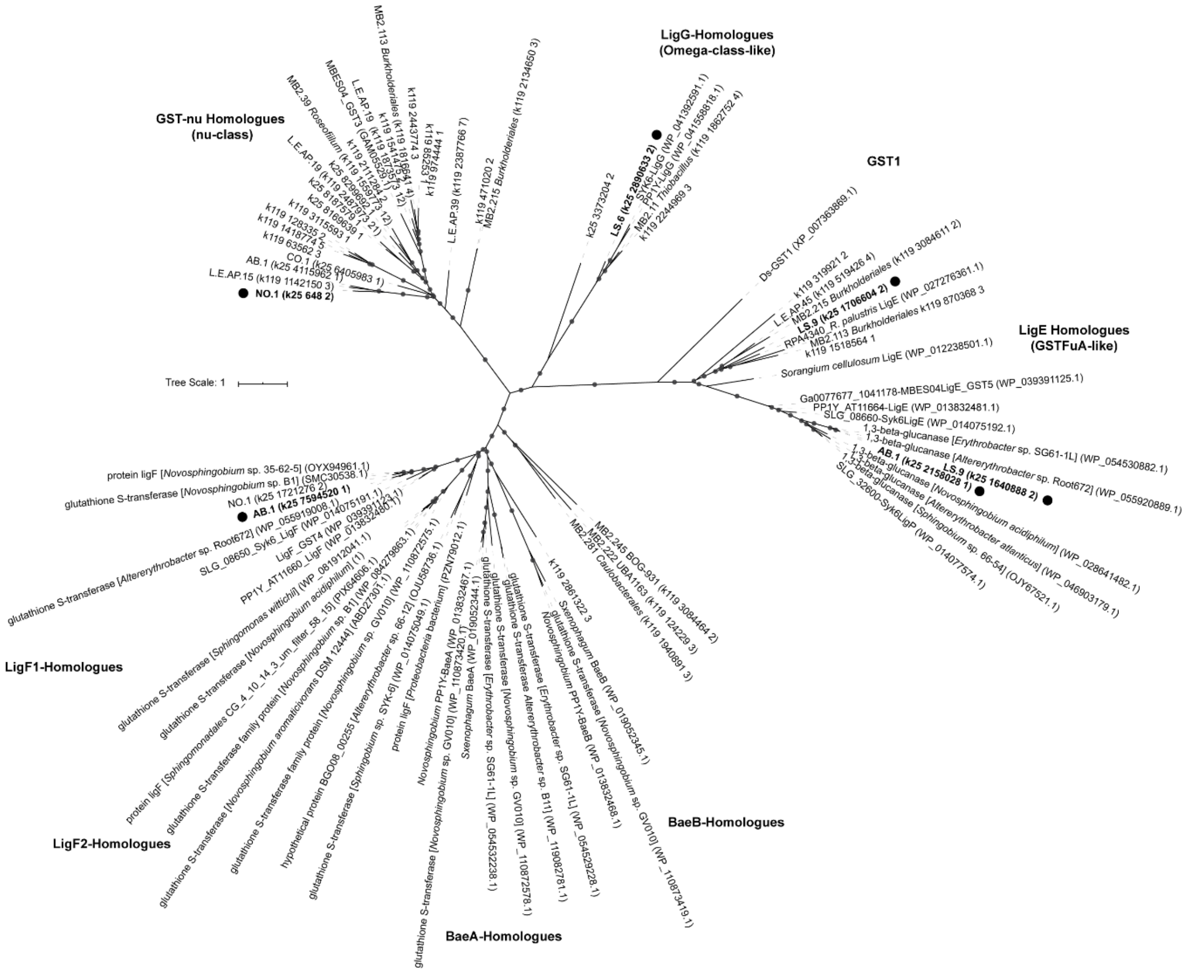


Supplemental Figure 14. Phylogenetic placement of assembled genes and transcripts into Glutathione S-Transferase tree from [1]. Amino acid sequences from metagenomics and metatranscriptome assemblies were aligned with reference sequences using MAFFT 7.407 using ginsi settings with a maximum of 1000 iterations, and phylogeny calculated using FastTree.

[1] Kontur, W. S. et al. "A heterodimeric glutathione *S*-transferase that stereospecifically breaks lignin's β(*R*)-aryl ether bond reveals the diversity of bacterial β-etherases." *Journal of Biological Chemistry* **294**, 1877-1890 (2019) [DOI: 10.1074/jbc.RA118.006548].
