# Supplementary material for: Genomics and metatranscriptomics of biogeochemical cycling and degradation of lignin-derived aromatic compounds in thermal swamp sediment
Source: ISME J. 2020 Nov 2;15(3):879–93. doi: 10.1038/s41396-020-00820-x (PMC8027834; doi:10.1038/s41396-020-00820-x)
Supplement: Supplementary file 2 — Supplemental Methods [file 41396_2020_820_MOESM2_ESM.pdf]

Genomics and metatranscriptomics of biogeochemical cycling and degradation of lignin-derived aromatic compounds in thermal swamp sediment

David J. Levy-Booth[1], Ameena Hashimi[1], Raphael Roccor[1], Liyang Liu[2], Scott Renneckar[2], Lindsay D. Eltis[1], William W. Mohn[1*]

1. Department of Microbiology and Immunology, The University of British Columbia, Vancouver, Canada
2. Department of Wood Science, The University of British Columbia, Vancouver, Canada

Running title: Aromatic catabolism in a thermal metatranscriptome

[*]To whom correspondence should be addressed: William W. Mohn, Department of Microbiology and Immunology, Life Sciences Institute, The University of British Columbia, 2350 Health Sciences Mall, Vancouver, BC, V6T 1Z3, Canada. Tel.: +1- 604–822–4285; Fax: +1-604-822-6041; E-mail: wmohn@mail.ubc.ca

Supplementary Methods

Additional sampling and 16S rRNA sequencing

About 500 ml of organic biofilms were sampled (3×) from Alpha and Epsilon pools, and moderately-warmed, sandy Melanic Brunisol soil (17°C) was collected adjacent to Delta pool. The V4 region of the prokaryotic small ribosomal subunit (16S rRNA) gene was amplified, prepared, sequenced using MiSeq, and analyzed using standard protocols (1–4) by Microbiome Insights Inc. (Vancouver, Canada).

Preparation of enzymatic mild acidolysis lignin (EMWL)

The wood chips were ground to pass a 20 mesh using a Wiley mill, following subsequent treatment to remove extractives based on an ASTM standard procedure (5,6). These extractive-free wood fiber was subjected to rotary ball milling (Retsch PM200) at 600 rpm for 6 hours at 10 min intervals with 5 min interval breaks. Ball milled wood powders was then suspended in pH 4.8 citrate buffer at a 2% concentration for enzymatic hydrolysis. In the mixture, 5% (w/w) cellulase and xylanase enzyme (Cellic CTec 3 and HTEE) and another 2% (w/v) Tween20 as a detergent and emulsifier were measured and added as described by Wu et al. (7). The mixture was then incubated in a shaker at 50 °C for 48 hrs, operating at 150 rpm. The enzymatically hydrolyzed residue, known as crude lignin, was filtered out, thoroughly washed with water, and freeze-dried. To further remove the sugar, 5 g dried crude lignins were extracted by 1000 ml aqueous acid dioxane (96:4 v/v, pH=2) at their boiling point (100 °C) for 2 hours under a flow of nitrogen. The mixture was separated by centrifugation. The supernatants, containing lignin in aqueous dioxane, was neutralized with sodium bicarbonate ($NaHCO_3$), and then added dropwise into 1000 ml acidic water (pH=2). The precipitated lignin was separated by filtration (0.45 µm PTFE membrane) and washed twice with acidic water and distilled water. The resultant enzymatic mill acidolysis lignin (EMAL) was dried and stored in a vacuum oven at 50 °C (8).

Lignin analysis

Lignin substrates were analyzed using ^{13}C nuclear magnetic resonance (NMR) and 2D heteronuclear single quantum coherence (HSQC) NMR analysis (9–11). Briefly, ~300 mg substrates underwent acetylation by dissolving them in pyridine/acetic anhydride (Fisher Sci.) (v, 1:1), washing thrice with 0.1 mol l^{-1} HCl, and vacuum drying prior to analysis. Acetylated and untreated lignin was dissolved in DMSO (Sigma Aldrich), mixed with 50 mg/ml chromium (III)

(Sigma Aldrich), and analyzed by 300 HMz NMR (Bruker Corp.) using acquisition parameters: 2000 scanning number, 1.4 s acquisition time, 2 s delay time, and 7 μs pulse length. The obtained spectrum were processed as in (12). The 13C-1H spectrum was acquired with following parameters: matrices of 4 data points for the 1H and 256 data points for 13C were collected with an interscan delay (D1) of 750 ms, 4 number of scans, and spectral width from 12.67 to -3.30 ppm. The obtained spectrum was refereed using the DMSO-d6 signal (2.50/39.5 ppm). Topspin 3.5 software was adopted to process the spectrum, including Fourier transform, baseline correction, and calibration. For semi-quantitative analysis, we defined the area of the G2 spectrum as the internal standard with a value of 100 aromatic units (100 Ar). All the other linkages and functional group were calculated as a number of 100 Ar.

Liquid chromatography

The thawed supernatant samples from the lignin incubations were acidified with acetic acid (10% final volume), centrifuged for 5 min at 21,000 x g and filtered (0.2 μm). Samples were analyzed by HPLC using an Agilent 1100 Series separations module equipped with a photodiode array detector. One-hundred μL of sample was injected onto a Kinetex EVO 5 μm C18 column (100 Å, 150 x 3.0 mm; Phenomenex, Torrance, U.S.A.), operated at a flow rate of 0.7 mL/min and equilibrated with aqueous 0.1% formic acid with 0.5% acetonitrile (buffer A). Buffer B contained 90% acetonitrile with 0.1% formic acid. The elution gradient was as following: 0-2 min, 0 to 10% buffer B; 2-15 min, 10 to 20% buffer B; 15-16 min, 20% buffer B; 16-17 min, 20 to 100% buffer B; 17-21 min, 100% buffer B; 21-22 min, 100 to 0% buffer B; 22-25 min, 0% buffer B. The detector was set at 280 nm. Authentic standards of 2,6-dimethoxy-1,4-benzoquinone, vanillic acid, syringic acid, vanillin, and syringealdehyde were used to identify monoaromatic compounds in the samples. Their retention times were 5.65 min, 6.12 min, 6.66 min, 7.39 min, and 8.28 min, respectively. Standard curves were obtained by injecting between 1 and 20 uM of authentic standards in 50 mM sodium phosphate buffer (dilutions derived from 100 mM stocks dissolved in DMSO).

Manual refinement of MAGs

High-quality bins (>90% completeness, < 10% contamination) were manually inspected for contig placement, gene content, and taxonomy. Misclustered contigs were identified with k-means clustering. Those that exhibited conflicting coverage patterns or contained duplicate SCGs

were removed from the final 39 assemblies, which corrected the taxonomy of some assemblies. For each bin, contig coverage across libraries was subject to average linkage hierarchical clustering, which was visualized alongside contig consensus taxonomy and presence of duplicate marker genes to manually identify contigs that had a high likelihood of being binned erroneously. These contigs were removed from final metagenome-assembled genomes (MAGs).

Custom HMM thresholds

To facilitate the identification aromatic compound degradation and anoxygenic photosystem genes, custom thresholds were set for 25 KEGG HMMs [Supplementary Data 1]. The genomes from this study and all 24,706 species-cluster representative genomes in the GTDB (13) were assessed using the 25 KEGG HMMs and HMMER 3.2.1 (http://hmmer.org/). For each HMM, hits with bitscores ≥ 20% of the default threshold value were aligned using MAFFT 7.407 (--auto), trimmed using TrimAl 1.2rev59 (-gt 0.30), and their phylogeny was calculated using FastTree 2.1.10 (--fastest).Phylogenetically modified HMM thresholds were set using the highest score obtained by proteins outside of the clade containing all significant hits, and these modified thresholds were used in the final analyses.

Calculating genomic abundance

Quality filtered read files from metagenome and transcriptome libraries were aligned to the 39 assembled genomes with BBMap at >97% identity. To capture potential biodiversity missed by the assembly approach, unmapped reads were subsequently aligned with the 351 remaining contig bins and 24,668 GTDB genomic fasta files. Reads mapping to multiple genomes were assigned as fractional counts. Genomes with >500 counts and >1% coverage were included in subsequent genomic analysis. Feature scaling was used to normalize counts in each metagenome library between 0 and 1 for all genomes independently to visualize the distribution of each genome across temperatures using the equation (1):

$$Counts_{norm.} = \frac{x - \min(x)}{\max(x) - \min(x)} \quad (1)$$

1. Apprill A, McNally S, Parsons R, Weber L. Minor revision to V4 region SSU rRNA 806R gene primer greatly increases detection of SAR11 bacterioplankton. Aquat Microb Ecol. 2015 Jun 4;75(2):129–37.

2. Caporaso JG, Lauber CL, Walters WA, Berg-Lyons D, Huntley J, Fierer N, et al. Ultra-high-throughput microbial community analysis on the Illumina HiSeq and MiSeq platforms. ISME J. 2012 Aug;6(8):1621–4.

3. Schloss PD, Westcott SL, Ryabin T, Hall JR, Hartmann M, Hollister EB, et al. Introducing mothur: Open-Source, Platform-Independent, Community-Supported Software for Describing and Comparing Microbial Communities. Appl Environ Microbiol. 2009 Dec 1;75(23):7537–41.

4. Kozich JJ, Westcott SL, Baxter NT, Highlander SK, Schloss PD. Development of a Dual-Index Sequencing Strategy and Curation Pipeline for Analyzing Amplicon Sequence Data on the MiSeq Illumina Sequencing Platform. Appl Environ Microbiol. 2013 Sep 1;79(17):5112–20.

5. A.S.T.M., Standard Test Method for Preparation of Extractive-Free Wood. 2013.

6. Liu, L.-Y.; Patankar, S. C.; Chandra, R. P.; Sathitsuksanoh, N.; Saddler, J. N.; Renneckar, S., Valorization of Bark Using Ethanol–Water Organosolv Treatment: Isolation and Characterization of Crude Lignin. *ACS Sustainable Chemistry & Engineering* **2020**.

7. Wu, J.; Chandra, R. P.; Kim, K. H.; Kim, C. S.; Pu, Y.; Ragauskas, A. J.; Saddler, J. N., Enhancing Enzyme-Mediated Hydrolysis of Mechanical Pulps by Deacetylation and Delignification. *ACS Sustainable Chemistry & Engineering* **2020,** *8* (15), 5847-5855.

8. ANDERSON GUERRA, I. F., LUCIAN A. LUCIA, AND DIMITRIS S. ARGYROPOULOS, Comparative Evaluation of Three Lignin Isolation Protocols for Various Wood Species. *Journal of agricultural and food chemistry* **2006,** *54*, 9696-9705.

9. Das L, Li M, Stevens J, Li W, Pu Y, Ragauskas AJ, et al. Characterization and Catalytic Transfer Hydrogenolysis of Deep Eutectic Solvent Extracted Sorghum Lignin to Phenolic Compounds. ACS Sustain Chem Eng. 2018 Aug 6;6(8):10408–20.

10. Balakshin MY, Capanema EA, Santos RB, Chang H, Jameel H. Structural analysis of hardwood native lignins by quantitative 13C NMR spectroscopy. Holzforschung. 2016 Feb 1;70(2):95–108.

11. Holtman KM, Chang H, Jameel H, Kadla JF. Quantitative 13C NMR Characterization of Milled Wood Lignins Isolated by Different Milling Techniques. J Wood Chem Technol. 2006 Apr 1;26(1):21–34.

12. Liu L-Y, Cho M, Sathitsuksanoh N, Chowdhury S, Renneckar S. Uniform Chemical Functionality of Technical Lignin Using Ethylene Carbonate for Hydroxyethylation and Subsequent Greener Esterification. ACS Sustain Chem Eng. 2018 Sep 4;6(9):12251–60.

13. Parks DH, Chuvochina M, Waite DW, Rinke C, Skarshewski A, Chaumeil P-A, et al. A standardized bacterial taxonomy based on genome phylogeny substantially revises the tree of life. Nat Biotechnol. 2018;36(10):996–1004.
